# Acetylcholinesterase and butyrylcholinesterase inhibitory activities of khellactone coumarin derivatives isolated from *Peucedanum japonicum* Thurnberg

**DOI:** 10.1038/s41598-020-78782-5

**Published:** 2020-12-10

**Authors:** Jeong Hyun Heo, Bo Hyun Eom, Hyung Won Ryu, Myung-Gyun Kang, Jong Eun Park, Doo-Young Kim, Jung-Hee Kim, Daeui Park, Sei-Ryang Oh, Hoon Kim

**Affiliations:** 1 Department of Pharmacy, and Research Institute of Life Pharmaceutical Sciences, Sunchon National University, Suncheon, 57922 Republic of Korea; 2 Natural Medicine Research Center, Korea Research Institute of Bioscience and Biotechnology, Cheong-ju si, Chungcheongbuk-do 28116 Republic of Korea; 3 Department of Predictive Toxicology, Korea Institute of Toxicology, Daejeon, 34114 Republic of Korea

**Keywords:** Biochemistry, Biological techniques, Chemical biology, Drug discovery

## Abstract

Cholinesterase (ChE) and monoamine oxidase (MAO) inhibitors have been attracted as candidate treatments for Alzheimer's disease (AD). Fifteen khellactone-type coumarins from the roots of *Peucedanum japonicum* Thunberg were tested for acetylcholinesterase (AChE), butyrylcholinesterase (BChE), and MAO inhibitory activities. Compound 3′-angeloyl-4′-(2-methylbutyryl)khellactone (**PJ13**) most potently inhibited AChE (IC_50_ = 9.28 µM), followed by 3′-isovaleryl-4′-(2-methylbutyroyl)khellactone (**PJ15**) (IC_50_ = 10.0 μM). Compound senecioyl-4′-angeloyl-khellactone (**PJ5**) most potently inhibited BChE (IC_50_ = 7.22 μM) and had the highest selectivity index (> 5.54), followed by 3′-senecioyl-4′-(2-methylbutyryl)khellactone (**PJ10**) and 3′,4′-disenecioylkhellactone (**PJ4**) (IC_50_ = 10.2 and 10.7 μM, respectively). Compounds **PJ13**, **PJ15**, and **PJ5** showed reversible and mixed-types of inhibition with K_i_ values of 5.98, 10.4 (for AChE), and 4.16 µM (for BChE), respectively. However, all 15 compounds weakly inhibited MAO-A and MAO-B. Molecular docking simulation revealed that **PJ13** had a higher binding affinity (− 9.3 kcal/mol) with AChE than **PJ15** (− 7.8 kcal/mol) or **PJ5** (− 5.4 kcal/mol), due to the formation of a hydrogen bond with Tyr121 (distance: 2.52 Å). On the other hand, the binding affinity of **PJ5** (− 10.0 kcal/mol) with BChE was higher than for **PJ13** (− 7.7 kcal/mol) or **PJ15** (− 8.1 kcal/mol), due to the formation of a hydrogen bond with Ser198 (distance: 2.05 Å). These results suggest that **PJ13** and **PJ5** are potential reversible selective inhibitors of AChE and BChE, respectively, for the treatment of AD.

## Introduction

Acetylcholinesterase (AChE) is a member of α/β hydrolase protein superfamily and breaks down an acetylcholine (ACh) into acetate and choline^1^. Alzheimer's disease (AD) is an age-associated memory/cognitive disorder, and its mechanism has not been determined, and no curative therapy has been developed^2^. Since cholinergic deficiency is present in AD, the relation between AChE and AD has been extensively studied^2,3^. AChE inhibitors (AChEIs) inhibit the hydrolysis of ACh (a neurotransmitter in the central nervous system), and as a result, increase ACh levels and ACh half-lives in autonomic ganglia and neuromuscular junctions, which are rich in ACh receptors^4^. AChEIs may be reversible or irreversible^5,6^. Commercially available AChEIs include piperidine-based (e.g., donepezil, Aricept)^7^, carbamate-based (rivastigmine, Exelon)^8^, phenanthrene-based (galantamine, Reminyl)^9^, and other inhibitors. The common potential side effects of AChEIs are diarrhea, headache, insomnia, nausea, and vomiting^10^. Butyrylcholinesterase (BChE) is mainly expressed in glial cells and white matter in the human brain, and as its name indicated, it breaks down butyrylcholine (BCh). BChE levels are significantly elevated in AD^11^, and in BChE knockout AD mice, a reported reduction in fibrin Aβ plaque by up to 70% suggests that BChE inhibition has therapeutic value^12^. Furthermore, AChE and BChE are known to be related to AD and to act independently of each other, which may lead to the diagnosis of disease and the development of potential drug targets^13^.

Recently dual- or multi-targeting inhibitors of acetylcholinesterase (AChE) and monoamine oxidase (MAO) have attracted research attention as candidate treatments for AD^14–19^. MAO catalyzes the oxidation of monoamines^20^, and has two isoforms (MAO-A and MAO-B). MAO was discovered almost a century ago and has been the subject of many structural, pharmacological, and biochemical studies on neurotransmitters^21^. MAO inhibitors (MAOIs) are currently used to treat depression^22^ and Parkinson's disease^23^, and several studies have concluded that MAOIs reduce Aβ plaque^24–26^, and thus, MAOIs are considered possible future treatments for AD^27^.

*Peucedanum japonicum* Thunberg is a herb found on the cliffs of islands in Korea, Japan, and the Philippines, and has traditionally been used to treat coughs, cramps, pain, rheumatism, asthma, and angina^28,29^. Furthermore, it has been shown to have anti-diabetic and anti-obesity^30,31^, anti-nociceptive^32^, anti-osteoporotic^33^, and anti-allergic lung inflammatory effects^34^. In traditional medicine, *P. japonicum* Thunberg is also believed to prevent stroke and vascular disease. On the other hand, an extract of *P. japonicum* Thunberg (KH020) has been reported to reduce Y-maze alternation behavior, and suggested to have therapeutic value for the prevention and treatment of vascular dementia^35^. From *P. japonicum* Thunberg, several compounds such as rutin, 3-*O*-caffeoylquinic acid, 4-*O*-caffeoylquinic acid, 5-*O*-caffeoylquinic acid, cnidioside A, praeroside II, praeroside III, apterin, esculin, (*R*)-peucedanol, and (*R*)-peucedanol 7-*O*-ß-d-glucopyranoside were identified^36^. In addition, a *P. japonicum* Thunberg extract was fractionated and found to contain a norisoprenoid glucoside, (3*S*)-*O*-ß-d-glucopyranosyl-6-[3-oxo-(2*S*)-butenylidenyl]-1,1,5-trimethylcyclohexan-(5*R*)-ol, and two phenylpropanoid glucosides, namely, 3-(2-*O*-ß-d-glucopyranosyl-4-hydroxyphenyl)-propanoic acid and methyl 3-(2-*O*-ß-d-glucopyranosyl-4-hydroxyphenyl) propanoate^37^. In another study, 80% EtOH was found to also contain peucedanol 7-*O*-β-d-glucopyranoside and myo-inositol^38^. Recently, khellactone coumarins were isolated from subfractions of *P. japonicum* roots by recycling HPLC, and reported to reduce NO levels in LPS-stimulated RAW264.7 cells and to inhibit anti-inflammatory response^39^.

However, little information is available about the anticholinergic actions of khellactone coumarins. Accordingly, we investigated the inhibitory effects of khellactone coumarins from *P. japonicum* Thunberg on AChE, BChE, and MAOs. In addition, we investigated the bindings and kinetics of the potent inhibitors senecioyl-4′-angeloyl-khellactone (**PJ5**), 3′-angeloyl-4′-(2-methylbutyryl)khellactone (**PJ13**), and 3′-isovaleryl-4′-(2-methylbutyroyl)khellactone (**PJ15**), and performed molecular docking simulations of these three compounds with AChE and BChE.

## Materials and methods

### Compounds

**Figure 1:**
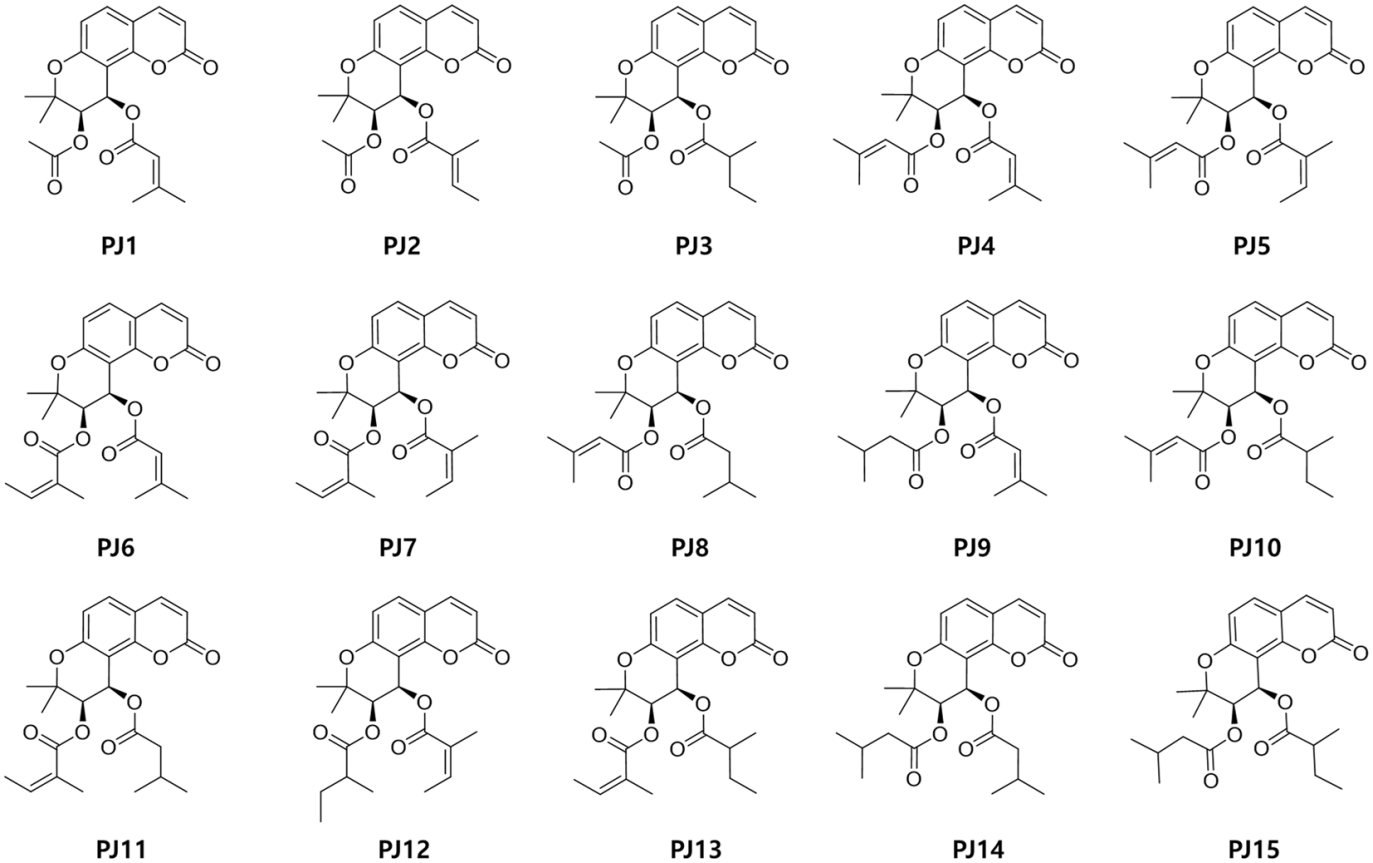
Chemical structures of khellactone coumarin derivatives from *Peucedanum japonicum* Thunberg^39^. **PJ1**, Isosamidin; **PJ2**, Pteryxin; **PJ3**, hyuganin; **PJ4**, 3′,4′-disenecioylkhellactone; **PJ5**, senecioyl-4′-angeloyl-khellactone; **PJ6**, calipteryxin; **PJ7**, anomalin; **PJ8**, 3′-senecioyl-4′-isovalerylkhellactone; **PJ9**, 3′-isovaleryl-4′-senecioylkhellactone; **PJ10**, 3′-senecioyl-4′-(2-methylbutyryl)khellactone; **PJ11**, 3′-isovaleryl-4′-angeloylkhellactone; **PJ12**, 3′-isovaleryl-4′-angeloylkhellactone; **PJ13**, 3′-angeloyl-4′-(2-methylbutyryl)khellactone; **PJ14**, 3′,4′-diisovalerylkhellactone; **PJ15**, 3′-isovaleryl-4′-(2-methylbutyroyl)khellactone.

Fifteen khellactone-type compounds were isolated from *P. japonicum* Thunberg (voucher specimen: PBC-484), and the structures were determined, as described previously^39^. Briefly, the dried roots of *P. japonicum* (5.0 kg) were extracted with 80% ethanol (EtOH) at room temperature three times to obtain 1.62 kg of solid extract. The 80% EtOH extract was further partitioned between *n*-hexane (114.2 g) and H_2_O (1.50 kg), and the *n*-hexane extract so obtained was subjected to preparative reverse phase chromatography (Xbridge Prep C_18_, 5 μm, Waters Corporation, Milford, MA, USA) using methanol (MeOH) and H_2_O (0–52.0 min, 66–88% MeOH; 52.0–53.0 min, 88–100% MeOH; 53.0–60.0 min, 100% MeOH). The fractions (Frs. 1–8) were collected and concentrated on a rotary evaporator under reduced pressure. Purification was conducted by recycling preparative HPLC. The yield of the khellactone-type coumarins obtained was ~ 1.5% from 80% EtOH extract determined by using ultra-performance liquid chromatography (UPLC) charged with photodiode array (PDA). Chemical structures of the compounds were identified by ^1^H NMR, ^13^C NMR, CD spectrum, UV spectrum, MS/MS, and HR-ESI–MS data (Supplementary Information 1) and their purities were determined by HPLC. The structures are shown in Fig. 1.

### Chemicals and enzymes

AChE (Type VI-S; from *Electrophorus electricus*), recombinant human MAO-A, MAO-B, and BChE (from equine serum), acetylthiocholine iodide (ATCI), kynuramine, benzylamine, *S*-butyrylthiocholine iodide (BTCI), 5,5′-dithiobis (2-nitrobenzoic acid) (DTNB), tacrine, donepezil, toloxatone, and lazabemide were purchased from Sigma-Aldrich (St. Louis, MO, USA). Clorgyline and pargyline (irreversible reference inhibitors of MAO-A and MAO-B, respectively) were from BioAssay Systems (Hayward, CA, USA)^40^.

### Enzyme assays

AChE assays were performed as described by Ellman et al.^41^ with slight modifications^42^. In brief, assays were performed using ~ 0.2 U/mL of AChE in the presence of 0.5 mM DTNB and 0.5 mM ACTI in 0.5 mL reaction mixtures, and continuously monitored for 10 min at 412 nm. DTNB was used for color development, caused by reaction between it and thiocholine (a product of AChE). For inhibitory assays, compounds were preincubated with AChE for 15 min prior to ATCI and DTNB addition. BChE activity was assayed using the same method, but BTCI was used instead of ATCI. MAO-A activity was continuously assayed using kynuramine (a substrate) at 316 nm for 20 min, and MAO-B activity was assayed using benzylamine at 250 nm for 30 min, as described previously^40^.

### Inhibitory activities and enzyme kinetics

Inhibitions of the activities of AChE, BChE, MAO-A, and MAO-B by the 15 compounds were investigated at an inhibitor concentration of 10 µM. IC_50_ values were also determined. The reference reversible inhibitors of AChE and BChE, MAO-A, and MAO-B used were tacrine (or donepezil), toloxatone and lazabemide, respectively, and the reference irreversible inhibitors of MAO-A and MAO-B used were clorgyline and pargyline, respectively. Kinetic parameters, inhibition types, and K_i_ values of **PJ5** (for BChE), **PJ13** and **PJ15** (for AChE) were determined as the methods previously described^43^. Enzyme kinetics were investigated at five different substrate concentrations, that is, at 0, ~ 1/2 × IC_50_, IC_50_, and 2 × IC_50_ for each inhibitor. The inhibition types and K_i_ values were determined using Lineweaver–Burk Plots and secondary plots.

### Analysis of inhibitor reversibilities

Inhibitor reversibilities were examined using the dialysis method^44^, using with AChE or BChE, rather than MAO enzymes. In brief, the experiment was performed by preincubating an inhibitor at ~ 2 × IC_50_ concentration with AChE or BChE for 30 min in 0.1 M sodium phosphate buffer (pH 7.2). Dialysis was conducted for 6 h with stirring and two buffer changes. Residual activities before (A_U_) and after (A_D_) dialysis were compared to those of non-treated controls, and reversibility types were determined by comparing A_D_ and A_U_ values.

### Docking simulations of PJ5, PJ13, and PJ15 with AChE or BChE

To simulate the dockings of **PJ5**, **PJ13**, and **PJ15** with AChE or BChE, we used Autodock Vina^45^, which has an automated docking facility. To define enzyme pockets, we used predefined active sites obtained from complexes between AChE and 3-[(1*S*)-1-(dimethylamino)ethyl]phenol (PDB ID: 1GQS) or donepezil (PDB ID: 6O4W), BChE and butyl-[(2 ~ {*S*})-1-(2-cycloheptylethylamino)-3-(1~{H}-indol-3-yl)-1-oxidanylidene-propan-2-yl]azanium (PDB ID: 6QAA), MAO-A and 7-methoxy-1-methyl-9*H*-β-carboline complex (PDB ID: 2Z5X), and MAO-B and pioglitazone complex (PDB ID: 4A79). To prepare **PJ5**, **PJ13**, and **PJ15** for docking simulation, ChemOffice program (http://www.cambridgesoft.com) was used to create the 2D structures of **PJ5**, **PJ13**, and **PJ15**, to convert them into 3D structures, and to perform energy minimizations. Docking simulations of the enzymes with **PJ5**, **PJ13**, and **PJ15** were performed using Chimera^46^. Based on the results of docking simulations, we checked for possible hydrogen bonding using bonding relaxation constraints of 0.4 Å and 20.0° using Chimera^47^.

### **Analysis of pharmacokinetic properties using in silico method**

 Drug-like properties of the lead compounds of **PJ5**, **PJ13**, and **PJ15** were analyzed using a web tool of SwissADME at http://www.swissadme.ch/^48^.

## Results

### Analysis of inhibitory activities

The structures and purities of the 15 compounds isolated from the *P. japonicum* Thunberg, were determined by 1D and 2D NMR spectra, UPLC-QTOF-MS analysis, and electronic circular dichroism spectra^39^. All were tested for AChE and BChE inhibitory activities at a concentration of 10 µM. **PJ13** and **PJ15** resulted in AChE residual activity of < 50% (Table 1). **PJ13** most potently inhibited AChE with an IC_50_ value of 9.28 µM, followed by **PJ15** and **PJ7** (IC_50_ = 10.0 and 17.9 µM, respectively). The other 12 compounds had IC_50_ values of ≥ 20 µM. In addition, four compounds resulted in BChE residual activity of < 50% (Table 1). **PJ5** most potently inhibited BChE with an IC_50_ value of 7.22 µM, followed by **PJ10 PJ4**, and **PJ9** (IC_50_ = 10.16, 10.66, 12.5 µM, respectively) (Table 1). The other 11 compounds had IC_50_ values of ≥ 40 µM. **PJ5** had the highest selectivity index of > 5.54. To examine the multi-targeting abilities of the compounds, we evaluated their inhibitory effects on MAO-A or MAO-B, which are auxiliary targets in AD. However, all compounds only weakly inhibited MAO-A or MAO-B with residual activities of > 63.1% at 10 µM (Table 1).

**Table 1:** Inhibitions of AChE, BChE, MAO-A, and MAO-B by khellactone coumarins from *Peucedanum japonicum* Thunberg roots.

Compounds	Residual activity at 10 µM (%)				IC_50_ (µM)		SI^a^
	AChE	BChE	MAO-A	MAO-B	AChE	BChE	
**PJ1**	92.1 ± 1.70	87.2 ± 1.21	86.0 ± 5.47	85.0 ± 0.57	> 40	> 40	
**PJ2**	73.4 ± 11.3	87.6 ± 7.14	85.4 ± 3.80	79.7 ± 3.47	> 40	> 40	
**PJ3**	97.2 ± 8.30	78.7 ± 4.03	81.2 ± 3.68	83.7 ± 2.33	> 40	> 40	
**PJ4**	85.4 ± 7.52	47.2 ± 1.95	100.3 ± 0.39	95.6 ± 4.80	21.3 ± 7.69	10.7 ± 0.060	1.99
**PJ5**	96.0 ± 2.83	34.9 ± 9.64	84.7 ± 9.30	96.0 ± 0.52	> 40	7.20 ± 0.79	> 5.56
**PJ6**	76.3 ± 6.46	84.3 ± 8.52	86.1 ± 1.93	63.1 ± 2.75	25.6 ± 4.50	> 40	< 0.64
**PJ7**	75.4 ± 5.72	69.9 ± 6.82	74.0 ± 1.16	73.8 ± 8.23	17.9 ± 5.59	> 40	< 0.45
**PJ8**	97.0 ± 1.40	76.8 ± 3.85	79.5 ± 2.05	68.5 ± 2.10	31.6 ± 4.40	> 40	< 0.79
**PJ9**	86.9 ± 9.92	50.9 ± 1.11	80.6 ± 5.02	75.0 ± 8.42	36.1 ± 0.66	12.5 ± 2.82	2.89
**PJ10**	86.4 ± 5.23	48.3 ± 2.78	92.1 ± 5.58	70.9 ± 2.61	> 40	10.2 ± 2.25	> 3.92
**PJ11**	88.1 ± 1.35	86.4 ± 3.20	75.4 ± 0.30	89.1 ± 1.36	> 40	> 40	
**PJ12**	58.3 ± 5.37	79.1 ± 3.32	78.9 ± 3.98	72.4 ± 3.70	29.0 ± 1.15	> 40	< 0.73
**PJ13**	48.0 ± 9.40	75.3 ± 1.77	88.5 ± 5.41	64.6 ± 8.82	9.28 ± 0.094	> 40	< 0.23
**PJ14**	51.5 ± 4.50	77.7 ± 0.80	77.0 ± 5.40	66.1 ± 5.23	28.1 ± 0.33	> 40	< 0.70
**PJ15**	50.0 ± 2.82	75.0 ± 6.24	76.9 ± 4.20	73.3 ± 7.21	10.0 ± 0.48	> 40	< 0.25
Toloxatone			1.08 ± 0.025^b^	–			
Lazabemide			–	0.063 ± 0.015 ^b^			
Clorgyline			0.007 ± 0.00070 ^b^	–			
Pargyline			–	0.028 ± 0.0043 ^b^			
Tacrine					0.27 ± 0.019	0.0087 ± 0.0009	31.0
Donepezil					0.0095 ± 0.0019	0.18 ± 0.0038	0.053

The values above are the means ± SEs of duplicate or triplicate experiments. Values for AChE and BChE were determined after preincubation of the enzymes with each compound for 15 min. ^a^ SI = IC_50_ of AChE/ IC_50_ of BChE, ^b^ IC_50_ value.

### Reversibilities of AChE and BChE inhibitions

**Figure 2:**
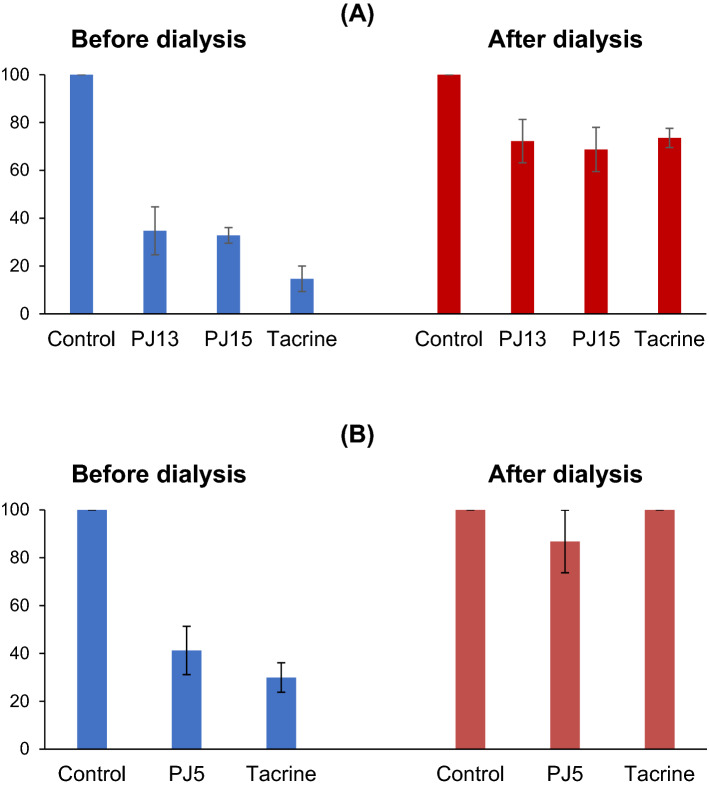
Recoveries of AChE inhibitions by **PJ13** and **PJ15** (**A**) and BChE inhibition by **PJ5** (**B**) after dialysis. Tacrine was used as the reference reversible inhibitor. The concentrations of the inhibitors used were ~ 2 × IC_50_: **PJ13**, 20 µM; **PJ15**, 20 µM; **PJ5**, 14 µM; and tacrine, 0.54 µM. For recovery experiments, preincubated enzyme mixtures were dialyzed as described in the text.

Inhibitory assays were carried out after preincubating AChE or BChE with inhibitors for 15 min. The reversibilities of AChE inhibitions by **PJ13** and **PJ15** were investigated using a dialysis-based method. Inhibitions of AChE by **PJ13** and **PJ15** recovered from 34.7% (A_U_) to 72.3% (A_D_) and from 32.8% to 68.7%, respectively, which were similar to those shown by tacrine (from 14.7% to 73.6%), a reversible AChE inhibitor (Fig. 2A). In addition, inhibition of BChE by **PJ5** recovered from 41.2% (A_U_) to 86.8% (A_D_), which was similar to that of tacrine (from 29.9% to 100%), also a reversible BChE inhibitor (Fig. 2B). These results indicate that **PJ13** and **PJ15** are reversible inhibitors of AChE and **PJ5** is a reversible inhibitor of BChE.

### Analysis of inhibitory patterns

**Figure 3:**
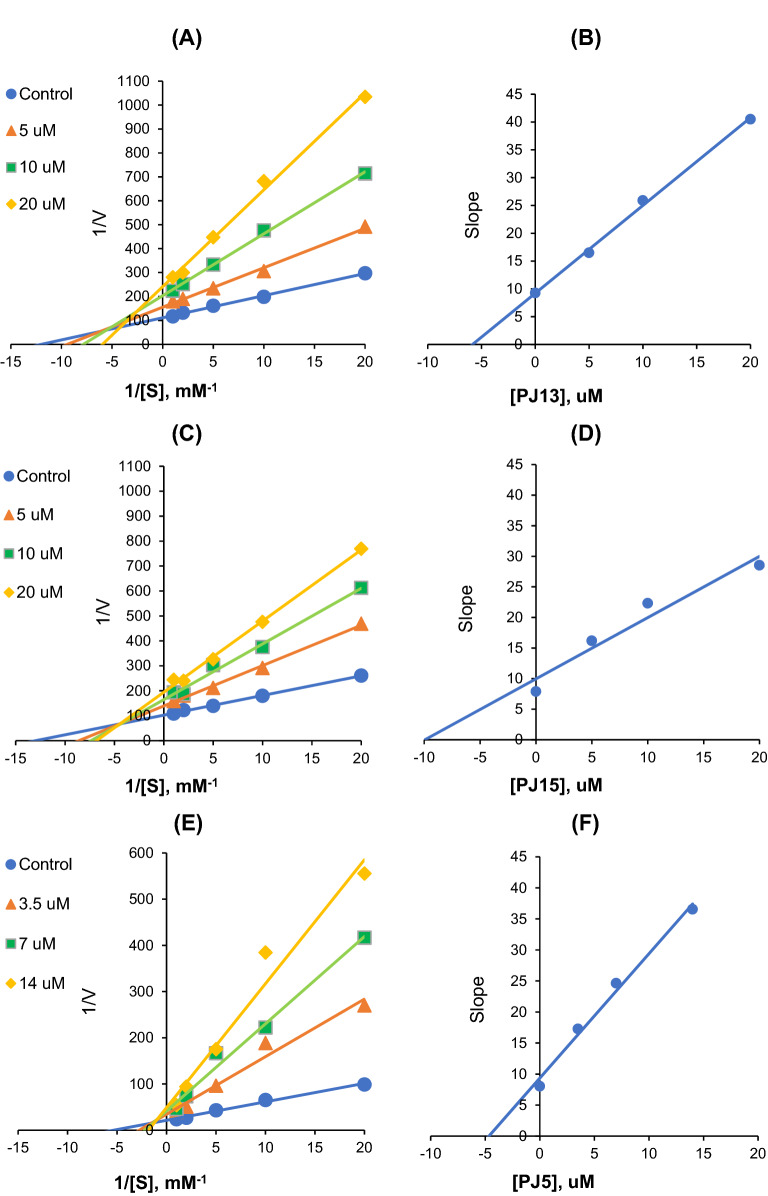
Lineweaver–Burk plots for the inhibitions of AChE by **PJ13** (**A**) and **PJ15** (**C**), and of BChE by **PJ5** (**E**), and respective secondary plots (**B**, **D**, **F**) of slopes against inhibitor concentration. Substrates were used at five different concentrations (0.05–1.0 mM). Experiments were carried out at three inhibitor concentrations at around their respective IC_50_ values. Initial reaction rates are expressed as increases in absorbance per min. K_m_ values of AChE and BChE were 0.1 and 0.18 mM, respectively.

Modes of AChE inhibitions by **PJ13** and **PJ15** were investigated using Lineweaver–Burk plots. Plots of AChE inhibition by **PJ13** were linear and lines intersected at a point, but not at the x- or y-axis (Fig. 3A). Secondary plots of the slopes of Lineweaver–Burk plots against inhibitor concentrations showed that the K_i_ value of **PJ13** for AChE inhibition was 5.99 ± 0.21 µM (Fig. 3B). Plots of AChE inhibitions by **PJ15** were also linear and did not intersect at the x- or y-axis (Fig. 3C), and the K_i_ value of **PJ15** for the AChE inhibition was 10.41 ± 0.67 μM (Fig. 3D). These results show **PJ13** and **PJ15** acted as mixed-type inhibitors of AChE. In addition, plots of BChE inhibition by **PJ5** were linear and intersected near the *y*-axis (Fig. 3E). Secondary plots showed the K_i_ value of **PJ5** for BChE inhibition was 4.16 ± 0.72 µM (Fig. 3F), showing **PJ5** acted as a mixed-type BChE inhibitor.

### Molecular docking simulation

**Figure 4:**
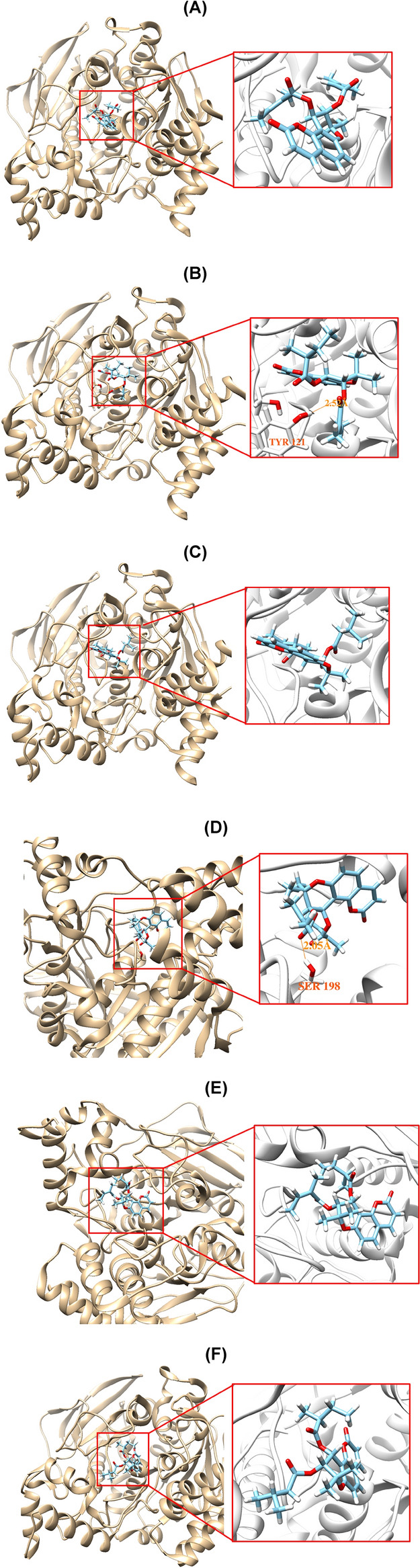
Docking simulations of **PJ5**, **PJ13**, and **PJ15** with AChE (1GQS) (**A**–**C**, respectively) and with BChE (6QAA) (**D**–**F**, respectively).

AutoDock Vina docking simulations showed that **PJ5**, **PJ13**, and **PJ15** located well at the binding site of 3-[(1*S*)-1-(dimethylamino)ethyl]phenol complexed with AChE and at that of butyl-[(2 ~ {*S*})-1-(2-cycloheptylethylamino)-3-(1 ~ {H}-indol-3-yl)-1-oxidanylidene-propan-2-yl]azanium complexed with BChE. The results of the docking simulation for AChE showed that **PJ13** interacted by forming a hydrogen bond with Tyr121 (distance: 2.52 Å). However, no hydrogen bond interaction was predicted for **PJ5** and **PJ15** (Fig. 4A–C). Docking simulation of **PJ5** with BChE implied that a hydrogen bonding interaction was established with Ser198 (distance: 2.05 Å) of BChE, whereas no hydrogen bond was proposed for **PJ13** and **PJ15** (Fig. 4D–F). The binding affinity of **PJ13** (− 9.3 kcal/mol) for AChE was higher than that of **PJ15** (− 7.8 kcal/mol) or **PJ5** (− 5.4 kcal/mol) (Table 2). In addition, **PJ5** had higher binding affinity for BChE (− 10.0 kcal/mol) than **PJ13** (− 7.7 kcal/mol) or **PJ15** (− 8.1 kcal/mol). The binding affinities of **PJ5**, **PJ13**, and **PJ15** with MAO-A or MAO-B were predicted to be weaker than those with AChE or BChE (Table 2). Docking simulations were provided in Supplementary Figure S17 (A–F). The binding score (− 4.8 kcal/mol) of **PJ13** for MAO-B was relatively higher than those of **PJ5** and **PJ15** in accordance with the residual activities at 10 µM.

**Table 2:** Binding energy values of **PJ5**, **PJ13**, and **PJ15** to AChE, BChE, MAO-A, and MAO-B.

Compounds	∆*G* (kcal/mol)			
	AChE	BChE	MAO-A	MAO-B
**PJ5**	− 5.4 (− 3.7)	− 10.0 (− 8.5)	1.3 (1.7)	0.3 (− 0.3)
**PJ13**	− 9.3 (− 8.6)	− 7.7 (− 6.5)	− 1.2 (− 1.6)	− 4.8 (− 4.8)
**PJ15**	− 7.8 (− 8.7)	− 8.1 (− 6.8)	− 0.6 (− 0.3)	− 3.4 (− 3.4)

The values in parentheses were obtained from the complexed or pre-defined structures with donepezil.

When the crystal structure of AChE complexed with donepezil (PDB ID: 6O4W) and the binding pockets for BChE, MAO-A, and MAO-B defined with donepezil were used for docking simulations, the binding scores of PJ compounds were similar to the values obtained with their complexed ligands (Tables 2 and Supplementary Table S3). From docking simulations with the AChE/donepezil complex (PDB ID: 6O4W), it was predicted that **PJ13** and **PJ15** formed one hydrogen bond with Tyr124 (distances = 2.602 and 2.994 Å, respectively), but **PJ5** did not form the bond. On the contrary, **PJ5** could form a hydrogen bond with Thr120 of BChE (distance = 3.354 Å), but **PJ13** and **PJ15** did not form (Supplementary Fig. S18).

### Pharmacokinetic properties using in silico method

From the SwissADME analysis, it was predicted that the lead compounds of **PJ5**, **PJ13**, and **PJ15** had high gastrointestinal adsorption abilities and cytochrome P450 inhibitory activities for 2C19, 2C9, and 3A4, however, they did not have blood–brain barrier (BBB) permeabilities (Table 3).

**Table 3:** Predicted pharmacokinetic properties of PJ5, PJ13, and PJ15.

Compounds	GI absorption	BBB permeant	P-gp substrate	CYP1A2 inhibitor	CYP2C19 inhibitor	CYP2C9 inhibitor	CYP2D6 inhibitor	CYP3A4 inhibitor	Log *K*_*p*_ (Skin permeation) (cm/s)
PJ5	High	No	No	No	Yes	Yes	No	Yes	− 5.64
PJ13	High	No	No	No	Yes	Yes	No	Yes	− 5.68
PJ15	High	No	No	No	Yes	Yes	Yes	Yes	− 5.65

GI, gastroinstestinal absorption; BBB, blood–brain barrier; P-gp, P-glycoprotein; CYP, cytochrome P450.

## Discussion

In this study, fifteen khellactone coumarin compounds from *P. japonicum* were analyzed for their abilities to inhibit AChE, BChE, MAO-A, and MAO-B. Compound **PJ13** (IC_50_ = 9.28 µM) most potently inhibited AChE, followed by **PJ15** and **PJ7** (10.0 and 17.9 µM, respectively), which indicated all three are highly potent natural AChE inhibitors, based on the IC_50_ values of < 20 µM^49^. The IC_50_ values of **PJ13** and **PJ15** were lower than those of the *C*–glucosylflavone, isovitexin-7-*O*-methyl ether (swertisin) (32.09 µg/mL, i.e., 71.9 µM) from *Anthocleista vogelii*^50^, the flavonoids tiliroside (23.5 µM) and quercetin (19.8 µM) from *Agrimonia pilosa*^51^, and the verbascosides decaffeoylverbascoside (16.1 µM) and acteoside (19.9 µM) from *Harpagophytum procumbens*^52^, but higher than those of sargachromanol I (SCI, 0.79 µM) from the brown alga *Sargassum siliquastrum* and dihydroberberine (1.18 µM) from *Coptis chinensis*^42^. Compared to other coumarin derivatives, the values of **PJ13** and **PJ15** were lower than those of scopoletin (52 µM) from *Vaccinium oldhami* Miquel^53^, a dihydropyranocoumarin decursinol (28 μM ) from *Angelica gigas* Nakai^54^, mansonone E (23.5 µM) from *Mansonia gagei*^55^, daphnetin (11.57 µM) from *Artemisia capillaris*^56^, and a furanocoumarin (R)-( +)-6′-hydroxy-7′-methoxybergamottin (11.2 µM) from *Citrus hystrix*^57^, and higher than those of esculetin (6.13 µM) from *A. capillaris*^56^, a dihydroxanthylectin-type coumarin 4′-hydroxy Pd–C-III (1.09 µM) from *Angelica decursiva*^58^_,_ and a 4-phenyl coumarin mesuagenin B (0.7 µM) from *Mesua elegans*^59^.

Regarding BChE inhibition, **PJ5** (IC_50_ = 7.22 µM) was the most potent inhibitor, followed by **PJ10** and **PJ4** (IC_50_ = 10.16 and 10.66 µM, respectively). The IC_50_ value of **PJ5** in this study was lower than those of broussonin A (7.50 µM) from *Anemarrhena asphodeloides*a^42^, isoacteoside (29.7 µM) from *H. procumbens*^52^, corenone B (10.9 μg/mL, i.e., 49.5 µM) from *Niphogeton dissecta*^60^, and kaempferol (62.5 µM) from *Cleistocalyx operculatus*^61^, but higher than that of 4′-hydroxy Pd–C-III (5.78 µM) from *A. decursiva*^58^. Compared to other coumarins, the IC_50_ value of **PJ5** for BChE inhibition was lower than those of hyuganin C (38.86 µM), from *Mutellina purpurea*^62^, a coumarin pteryxin (12.96 μg/mL, i.e., 33.5 µM) from *M. purpurea*^63^, the esculetin (9.29 µM) and the daphnetin (8.66 µM)^56^, and it might be concluded that **PJ5** is the most potent BChE inhibitor in natural coumarins reported.

These results show that **PJ5** is potent and selective inhibitor of BChE, and that **PJ13** and **PJ15** are selective inhibitors of AChE. It might be suggested that combination of compounds effectively inhibit ChE. The possibility of dual inhibition of AChE and MAO enzymes was investigated for dual- or multi-targeting therapeutic purposes in AD^15,17–19^. However, in the present study, no tested khellactone coumarin showed dual inhibitory activity.

Structurally, **PJ5**, **PJ13,** and **PJ15** contain a coumarin ring system, and the coumarins are known to have a variety of biological functions, which include anti-inflammatory, anticancer, antiviral, antioxidant, and antidepressant effects, and some have been shown to inhibit AChE and BChE^58,64^. **PJ13** and **PJ15** differ structurally as different substituents are bound to the 3C ester. **PJ13** [(9*R*,10*R*)-8,8-dimethyl-10-((2-methylbutanoyl)oxy)-2-oxo-9,10-dihydro-2*H*,8*H*-pyrano[2,3-f]chromen-9-yl (*E*)-2-methylbut-2-enoate] had a substituent [(*Z*)-but-2-en-2-yl] with a double bond between 1 and 2C in the *sec*-butyl structure, whereas **PJ15** [(9*R*,10*R*)-8,8-dimethyl-10-((2-methylbutanoyl)oxy)-2-oxo-9,10-dihydro-2*H*,8*H*-pyrano[2,3-f]chromen-9-yl 3-methylbutanoate] has an isobutyl group in this position. The AChE inhibitory activity of **PJ15** was slightly higher than that of **PJ13**, which contains a (*Z*)-but-2-en-2-yl group. **PJ4**, **PJ5,** and **PJ10** share a common 3-methylbut-2-enoate structure, and showed relatively higher BChE activities than other compounds. The higher BChE inhibitory activity of **PJ9** than **PJ8** appeared to be due to the different position of the double bond.

AChE or BChE inhibitors have been reported to exhibit competitive, noncompetitive, and mixed-type inhibitory patterns^42,58^. In the present study, potent inhibitions of AChE by **PJ13** and **PJ15** and of BChE by **PJ5** were found to be reversible and to exhibit mixed-type inhibition, with K_i_ values of 5.98, 10.4, and 4.16 µM, respectively. These results suggest that **PJ13**, **PJ15**, and **PJ5** bind to the allosteric site or the substrate-binding site of AChE.

Docking simulation analysis with AChE revealed that the **PJ13** interacted with the phenolic hydroxyl group of Tyr121 to form a hydrogen bond, while no hydrogen-bond was predicted for **PJ5** and **PJ15**. In addition, the oxygen of the carboxyl group of **PJ5** formed a hydrogen bond with Ser198 of BChE, whereas no hydrogen bonding was suggested for **PJ13** and **PJ15**. These results imply that the existence of the hydrogen bond in the complex has major effects on binding energies. Furthermore, the results concur with the K_i_ values and binding affinities of AChE or BChE for **PJ5**, **PJ13**, or **PJ15**.

To explain the reason **PJ15** inhibits AChE more selectively than **PJ5**, Van der Waals (VDW) distances and interactions were examined at C16, C17, C18, and C19 (for PJ15) or C21 (for **PJ5**) atoms in the docked ligands, according to the difference between **PJ15** and **PJ5**, i.e., the 2-methyl-butane and the 2-methyl-butene group, respectively (Figs. 1 and Supplementary Fig. S16). It was predicted that thirteen and five VDW interactions were formed with **PJ15** and **PJ5**, respectively, within a distance of 4 Å (Supplementary Table S1 and S2). The VDW interactions of **PJ15** could inhibit AChE more selectively than **JP5**.

In molecular dynamics analysis, average root mean square deviation (RMSD) values of **PJ5**, **PJ13**, and **PJ15** for AChE were estimated to be 0.767, 0.684, and 0.752 Å, respectively, and those for BChE were 0.738, 0.823, 0.757 Å, respectively (Supplementary Figure S19). The results supported well the experimental data and the docking simulations in this study.

In a previous study, it was observed that **PJ5**, **PJ13**, and **PJ15** were non-toxic up to 10 µg/µL (i.e., ~ 25 mM) and exhibited potent for anti-inflammatory effects at 10 µg/µL in previous study^39^, which suggests **PJ5**, **PJ13**, and **PJ15** be considered candidates for the treatment of AD as ChE inhibitors with anti-inflammatory activities.

## Conclusion

Among the fifteen khellactone coumarin compounds isolated from *P. japonicum* Thunberg, **PJ5** and **PJ13** were found to potently and effectively inhibited BChE and AChE, respectively. Furthermore, these inhibitors were reversible and caused by mixed inhibition. Molecular docking simulations showed that **PJ13** had the highest binding affinity for AChE at − 9.3 kcal/mol, and that **PJ5** had the highest binding affinity for BChE at − 10.0 kcal/mol. These results supported the notion that **PJ13** and **PJ5** should be considered novel, potent, and selective inhibitors of AChE and BChE, respectively. In addition, our findings suggest that **PJ5**, **PJ13**, and **PJ15** are nontoxic, reversible AChE and BChE inhibitors and candidates for the treatment of AD.

## Supplementary information

Supplementary Information 1.
