# Supplementary material for: Acetylcholinesterase and butyrylcholinesterase inhibitory activities of khellactone coumarin derivatives isolated from Peucedanum japonicum Thurnberg
Source: Sci Rep. 2020 Dec 10;10:21695. doi: 10.1038/s41598-020-78782-5 (PMC7730441; doi:10.1038/s41598-020-78782-5)
Supplement: Supplementary file 1 — Supplementary Information 1. [file 41598_2020_78782_MOESM1_ESM.docx]

**Supporting Information**

**Acetylcholinesterase and butyrylcholinesterase inhibitory activities of khellactone coumarin derivatives isolated from *Peucedanum japonicum* Thurnberg**

Jeong Hyun Heo^1,#^, Bo Hyun Eom^1,#^, Hyung Won Ryu^2^, Myung-Gyun Kang^3^, Jong Eun Park^1^, Doo-Young Kim^2^, Jung-Hee Kim^2^, Daeui Park^3^, Sei-Ryang Oh^2^, Hoon Kim^1🖂^

^1^ Department of Pharmacy, and Research Institute of Life Pharmaceutical Sciences, Sunchon National University, Suncheon 57922, Republic of Korea

^2^ Natural Medicine Research Center, Korea Research Institute of Bioscience and Biotechnology, Cheong-ju si, Chungcheongbuk-do, 28116, Republic of Korea

^3^ Department of Predictive Toxicology, Korea Institute of Toxicology, Daejeon 34114, Republic of Korea

**Table of Contents**

**Table S1.** The VDW distances and interactions of **PJ15** with AChE (1GQS) 5

**Table S2.**The VDW distances and interactions of **PJ5** with AChE (1GQS) 5

**Table S3.** Binding scores of PJ compounds to target proteins with donepezil. 6

**Figure S1.1.** ^1^H NMR spectrum of (+) 3'-acetoxy-4'-senecioyloxykhellactone (**1**) 7

**Figure S1.2.** ^13^C NMR spectrum of (+) 3'-acetoxy-4'-senecioyloxykhellactone (**1**) 7

**Figure S1.3.** CD spectrum of (+) 3'-acetoxy-4'-senecioyloxykhellactone (**1**) 8

**Figure S1.4.** UV spectrum and MS/MS spectrum of (+) 3'-acetoxy-4'-senecioyloxykhellactone (**1**) 9

**Figure S1.5.** HR-ESI-MS data of (+) 3'-acetoxy-4'-senecioyloxykhellactone (**1**) 9

**Figure S2.1.** ^1^H NMR spectrum of (+) 3'-acetoxy-4'-tigloylkhellacton (**2**) 10

**Figure S2.2.** ^13^C NMR spectrum of (+) 3'-acetoxy-4'-tigloylkhellacton (**2**) 10

**Figure S2.3.** CD spectrum of (+) 3'-acetoxy-4'-tigloylkhellacton (**2**) 11

**Figure S2.4.** UV spectrum and MS/MS spectrum of (+) 3'-acetoxy-4'-tigloylkhellacton (**2**)12

**Figure S2.5.** HR-ESI-MS data of (+) 3'-acetoxy-4'-tigloylkhellacton (**2**) 12

**Figure S3.1.** ^1^H NMR spectrum of (+) 3'-acetoxy-4'-(2-methylbutyroyl)khellactone (**3**) 13

**Figure S3.2.** ^13^C NMR spectrum of (+) 3'-acetoxy-4'-(2-methylbutyroyl)khellactone (**3**) 13

**Figure S3.3.** CD spectrum of (+) 3'-acetoxy-4'-(2-methylbutyroyl)khellactone (**3**) 14

**Figure S3.4.** UV spectrum and MS/MS spectrum of (+) 3'-acetoxy-4'-(2-methylbutyroyl) khellactone (**3**) 15

**Figure S3.5.** HR-ESI-MS data of (+) 3'-acetoxy-4'-(2-methylbutyroyl)khellactone (**3**) 15

**Figure S4.1.** ^1^H NMR spectrum of (-) 3', 4'-di senecioylkhellactone (**4**) 16

**Figure S4.2.** ^13^C NMR spectrum of (-) 3', 4'-di senecioylkhellactone (**4**) 16

**Figure S4.3.** CD spectrum of (-) 3', 4'-di senecioylkhellactone (**4**) 17

**Figure S4.4.** UV spectrum and MS/MS spectrum of (-) 3', 4'-di senecioylkhellactone (**4**) 18

**Figure S4.5.** HR-ESI-MS data of (-) 3', 4'-di senecioylkhellactone (**4**) 18

**Figure S5.1.** ^1^H NMR spectrum of (-) 3'-senecioyl-4'-angeloylkhellactone (**5**) 19

**Figure S5.2.** ^13^C NMR spectrum of (-) 3'-senecioyl-4'-angeloylkhellactone (**5**) 19

**Figure S5.3.** CD spectrum of (-) 3'-senecioyl-4'-angeloylkhellactone (**5**) 20

**Figure S5.4.** UV spectrum and MS/MS spectrum of (-) 3'-senecioyl-4'-angeloylkhellactone (**5**) 21

**Figure S5.5.** HR-ESI-MS data of (-) 3'-senecioyl-4'-angeloylkhellactone (**5**) 21

**Figure S6.1.** ^1^H NMR spectrum of (+) 3'-angeloyl-4'-senecioylkhellactone (**6**) 22

**Figure S6.2.** ^13^C NMR spectrum of (+) 3'-angeloyl-4'-senecioylkhellactone (**6**) 22

**Figure S6.3.** CD spectrum of (+) 3'-angeloyl-4'-senecioylkhellactone (**6**) 23

**Figure S6.4.** UV spectrum and MS/MS spectrum of (+) 3'-angeloyl-4'-senecioylkhellactone (**6**) 24

**Figure S6.5.** HR-ESI-MS data of (+) 3'-angeloyl-4'-senecioylkhellactone (**6**) 24

**Figure S7.1.** ^1^H NMR spectrum of (+) 3', 4'-diangeloylkhellactone (**7**) 25

**Figure S7.2.** ^13^C NMR spectrum of (+) 3', 4'-diangeloylkhellactone (**7**) 25

**Figure S7.3.** CD spectrum of (+) 3', 4'-diangeloylkhellactone (**7**) 26

**Figure S7.4.** UV spectrum and MS/MS spectrum of (+) 3', 4'-diangeloylkhellactone (**7**) 27

**Figure S7.5.** HR-ESI-MS data of (+) 3', 4'-diangeloylkhellactone (**7**) 27

**Figure S8.1.** ^1^H NMR spectrum of (-) 3'-senecioyl-4'-isovalerylkhellactone (**8**) 28

**Figure S8.2.** ^13^C NMR spectrum of (-) 3'-senecioyl-4'-isovalerylkhellactone (**8**) 28

**Figure S8.3.** CD spectrum of (-) 3'-senecioyl-4'-isovalerylkhellactone (**8**) 29

**Figure S8.4.** UV spectrum and MS/MS spectrum of (-) 3'-senecioyl-4'-isovalerylkhellactone (**8**) 30

**Figure S8.5.** HR-ESI-MS data of (-) 3'-senecioyl-4'-isovalerylkhellactone (**8**) 30

**Figure S9.1.** ^1^H NMR spectrum of (-) 3'-isovaleryl-4'-senecioylkhellactone (**9**) 31

**Figure S9.2.** ^13^C NMR spectrum of (-) 3'-isovaleryl-4'-senecioylkhellactone (**9**) 31

**Figure S9.3.** CD spectrum of (-) 3'-isovaleryl-4'-senecioylkhellactone (**9**) 32

**Figure S9.4.** UV spectrum and MS/MS spectrum of (-) 3'-isovaleryl-4'-senecioylkhellactone (**9**) 33

**Figure S9.5.** HR-ESI-MS data of (-) 3'-isovaleryl-4'-senecioylkhellactone (**9**) 33

**Figure S10.1.** ^1^H NMR spectrum of (-) 3'-senecioyl-4'-(2-methylbutyroyl) khellactone (**10**) 34

**Figure S10.2.** ^13^C NMR spectrum of (-) 3'-senecioyl-4'-(2-methylbutyroyl) khellactone (**10**) 34

**Figure S10.3.** CD spectrum of (-) 3'-senecioyl-4'-(2-methylbutyroyl) khellactone (**10**) 35

**Figure S10.4.** UV spectrum and MS/MS spectrum of (-) 3'-senecioyl-4'-(2-methylbutyroyl) khellactone (**10**) 36

**Figure S10.5.** HR-ESI-MS data of (-) 3'-senecioyl-4'-(2-methylbutyroyl) khellactone (**10**) 36

**Figure S11.1.** ^1^H NMR spectrum of (-) 3'-isovaleryl-4'-angeloylkhellactone (**11**) 37

**Figure S11.2.** ^13^C NMR spectrum of (-) 3'-isovaleryl-4'-angeloylkhellactone (**11**) 37

**Figure S11.3.** CD spectrum of (-) 3'-isovaleryl-4'-angeloylkhellactone (**11**) 38

**Figure S11.4.** UV spectrum and MS/MS spectrum of (-) 3'-isovaleryl-4'-angeloylkhellactone (**11**) 39

**Figure S11.5.** HR-ESI-MS data of (-) 3'-isovaleryl-4'-angeloylkhellactone (**11**) 40

**Figure S12.1.** ^1^H NMR spectrum of (-) 3'-(2-methylbutyryl)-4'-angeloylkhellactone (**12**) 40

**Figure S12.2.** ^13^C NMR spectrum of (-) 3'-(2-methylbutyryl)-4'-angeloylkhellactone (**12**) 40

**Figure S12.3.** CD spectrum of (-) 3'-(2-methylbutyryl)-4'-angeloylkhellactone (**12**) 41

**Figure S12.4.** UV spectrum and MS/MS spectrum of (-) 3'-(2-methylbutyryl)-4'-angeloyl khellactone (**12**) 42

**Figure S12.5.** HR-ESI-MS data of (-) 3'-(2-methylbutyryl)-4'-angeloylkhellactone (**12**) 42

**Figure S13.1.** ^1^H NMR spectrum of (+) 3'-angeloyl-4'-(2-methylbutyryl)khellactone (**13**) 43

**Figure S13.2.** ^13^C NMR spectrum of (+) 3'-angeloyl-4'-(2-methylbutyryl)khellactone (**13**) 43

**Figure S13.3.** CD spectrum of (+) 3'-angeloyl-4'-(2-methylbutyryl)khellactone (**13**) 44

**Figure S13.4.** UV spectrum and MS/MS spectrum of (+) 3'-angeloyl-4'-(2-methylbutyryl) khellactone (**13**) 45

**Figure S13.5.** HR-ESI-MS data of (+) 3'-angeloyl-4'-(2-methylbutyryl)khellactone (**13**) 45

**Figure S14.1.** ^1^H NMR spectrum of (-) 3',4'-diisovalerylkhellactone (**14**) 46

**Figure S14.2.** ^13^C NMR spectrum of (-) 3',4'-diisovalerylkhellactone (**14**) 46

**Figure S14.3.** CD spectrum of (-) 3',4'-diisovalerylkhellactone (**14**) 47

**Figure S14.4.** UV spectrum and MS/MS spectrum of (-) 3',4'-diisovalerylkhellactone (**14**) 48

**Figure S14.5.** HR-ESI-MS data of (-) 3',4'-diisovalerylkhellactone (**14**) 48

**Figure S15.1.** ^1^H NMR spectrum of (-) 3'-isovaleryl-4'-(2-methylbutyryl)khellactone (**15**) 49

**Figure S15.2.** ^13^C NMR spectrum of (-) 3'-isovaleryl-4'-(2-methylbutyryl)khellactone (**15**) 49

**Figure S15.3.** CD spectrum of (-) 3'-isovaleryl-4'-(2-methylbutyryl)khellactone (**15**) 50

**Figure S15.4.** UV spectrum and MS/MS spectrum of (-) 3'-isovaleryl-4'-(2-methylbutyryl) khellactone (**15**) 51

**Figure S15.5.** HR-ESI-MS data of (-) 3'-isovaleryl-4'-(2-methylbutyryl)khellactone (**15**) 51

**Figure S16.** Structures of **PJ15** and **PJ5** with the carbon numbers of 2-methyl-butane and 2-methyl-butene group, respectively. 52

**Figure S17.** Docking simulations of **PJ5**, **PJ13**, and **PJ15** with MAO-A (2Z5X) (A-C, respectively) and MAO-B (4A79) (D-F, respectively).. 53

**Figure S18.** Docking simulations of **PJ13** (A) and **PJ15** (B) with AChE (PDB ID: 6O4W) complexed with donepezil and **PJ5** (C) with BChE (PDB ID: 6QAA) pre-defined with donepezil. 55

**Figure S19.** Plots of root mean square deviation during 100 ps MD simulation of AChE (A) BChE (B) in complexes with **PJ5**, **PJ13**, and **PJ15**... 56

**Table S1.** The VDW distances and interactions of **PJ15** with AChE (1GQS)

| Atom in Compound | Amino Acid Residue | Atom in Residue | Overlap | Distance |
| --- | --- | --- | --- | --- |
| C16 | Tyr130 | OH | -0.004 | 3.344 |
| C16 | Gly117 | O | -0.352 | 3.652 |
| C17 | Gly117 | O | -0.209 | 3.509 |
| C17 | Gly117 | C | -0.073 | 3.563 |
| C17 | Trp84 | CE3 | -0.032 | 3.672 |
| C17 | Gly117 | N | -0.385 | 3.905 |
| C17 | Trp84 | CZ3 | -0.272 | 3.912 |
| C18 | Gly117 | O | -0.113 | 3.413 |
| C18 | Tyr130 | CZ | -0.242 | 3.732 |
| C19 | Leu127 | CD2 | -0.147 | 3.907 |
| C19 | Gly123 | C | -0.292 | 3.782 |
| C19 | Tyr130 | CE2 | -0.310 | 3.950 |

**Table S2.** The VDW distances and interactions of **PJ5** with AChE (1GQS)

| Atom in Compound | Amino Acid Residue | Atom in Residue | Overlap | Distance |
| --- | --- | --- | --- | --- |
| C16 | Ser122 | OG | -0.394 | 3.464 |
| C18 | Tyr121 | CE1 | -0.316 | 3.836 |
| C21 | Tyr70 | CD2 | -0.245 | 3.885 |
| C21 | Asp72 | CB | -0.16 | 3.92 |
| C21 | Ser122 | CB | -0.24 | 4.00 |

**Table S3**. Binding scores of PJ compounds to target proteins with donepezil.

| Compounds | Binding Affinity (kcal/mol) | | | |
| --- | --- | --- | --- | --- |
|  | AChE (6O4W) | BChE (6QAA) | MAO-A (2Z5X) | MAO-B (4A79) |
| **PJ1** | -4.9 | -6.6 | -1.7 | -2.4 |
| **PJ2** | -5.5 | -5.2 | -3.0 | -2.5 |
| **PJ3** | -4.6 | -5.5 | -2.6 | -2.7 |
| **PJ4** | -6.4 | -8.5 | 0.1 | -1.2 |
| **PJ5** | **-3.7** | **-8.5** | 1.7 | -0.3 |
| **PJ6** | -6.5 | -5.8 | -1.4 | -5.3 |
| **PJ7** | -6.7 | -5.8 | -0.2 | -2.5 |
| **PJ8** | -5.9 | -5.6 | 0.3 | -3.7 |
| **PJ9** | -5.6 | -8.1 | -0.2 | -3.3 |
| **PJ10** | -5.6 | -8.4 | 0.4 | -2.9 |
| **PJ11** | -5.4 | -4.9 | -1.3 | -1.0 |
| **PJ12** | -6.8 | -5.9 | 0.2 | -3.6 |
| **PJ13** | **-8.6** | **-6.5** | -1.6 | -4.8 |
| **PJ14** | -6.8 | -6.3 | 0.1 | -3.7 |
| **PJ15** | **-8.7** | **-6.8** | -0.3 | -3.4 |
| Donepezil | -11.2 | -9.4 | -2.8 | -10.3 |


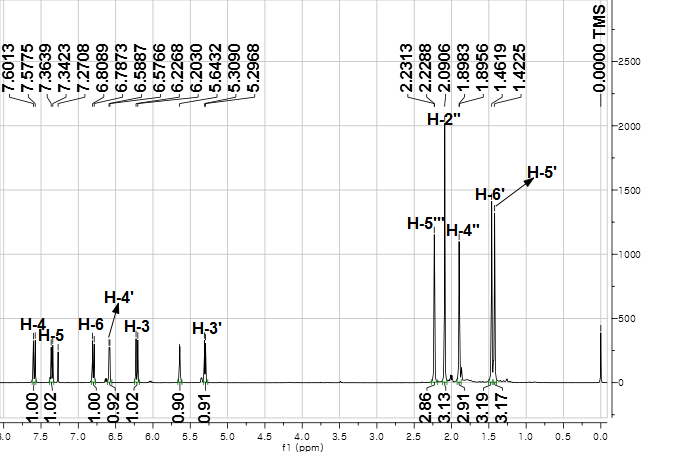


**Figure S1.1.** ^1^H NMR spectrum of (+) 3'-acetoxy-4'-senecioyloxykhellactone (**1**).


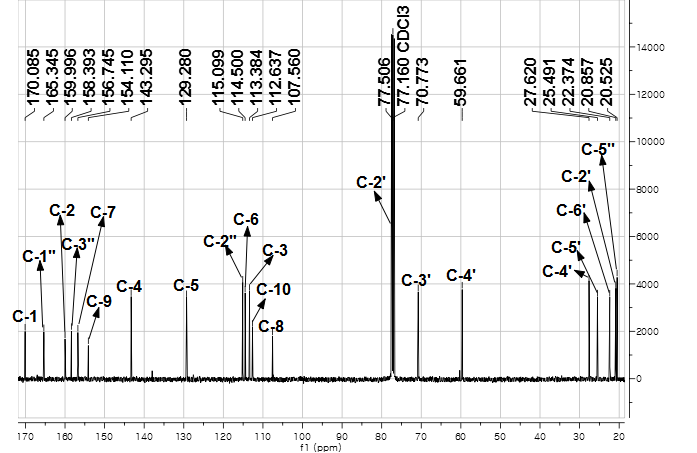


**Figure S1.2.** ^13^C NMR spectrum of (+) 3'-acetoxy-4'-senecioyloxykhellactone (**1**).

**
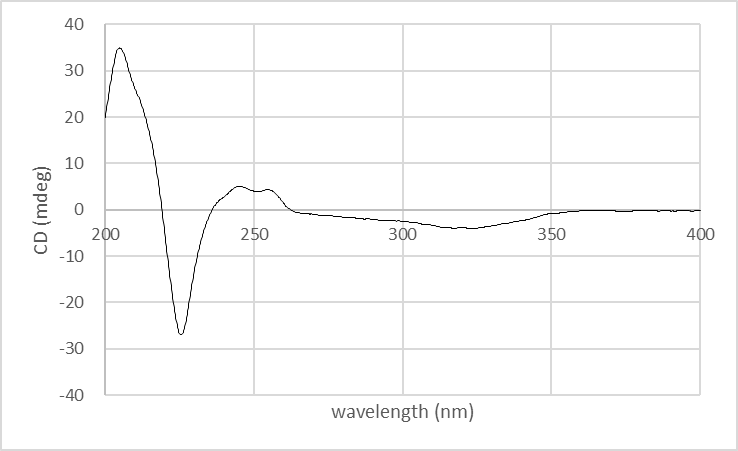
**

**Figure S1.3.** CD spectrum of (+) 3'-acetoxy-4'-senecioyloxykhellactone (**1**).


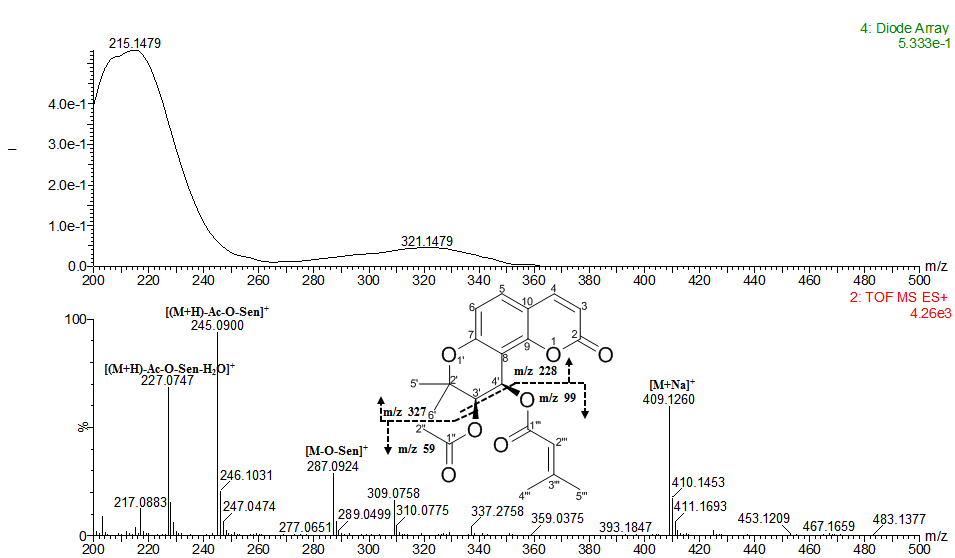


**Figure S1.4.** UV spectrum and MS/MS spectrum of (+) 3'-acetoxy-4'-senecioyloxykhellactone (**1**).


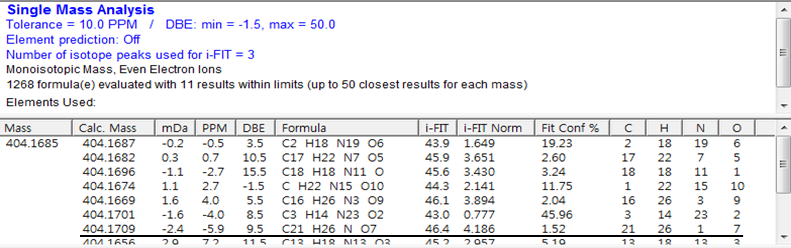


**Figure S1.5.** HR-ESI-MS data of (+) 3'-acetoxy-4'-senecioyloxykhellactone (**1**).


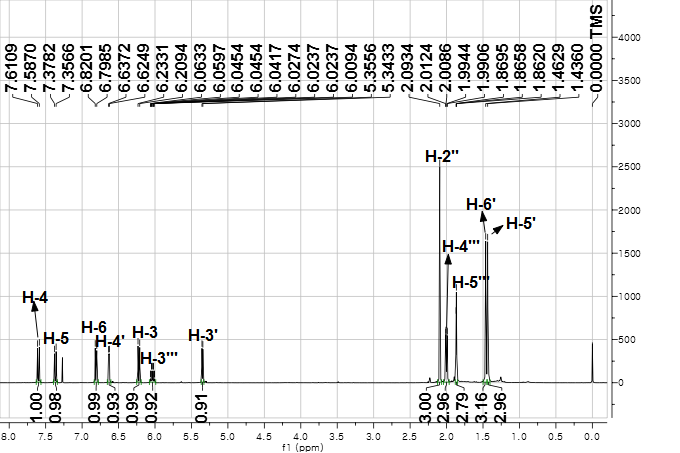


**Figure S2.1.** ^1^H NMR spectrum of (+) 3'-acetoxy-4'-tigloylkhellacton (**2**).


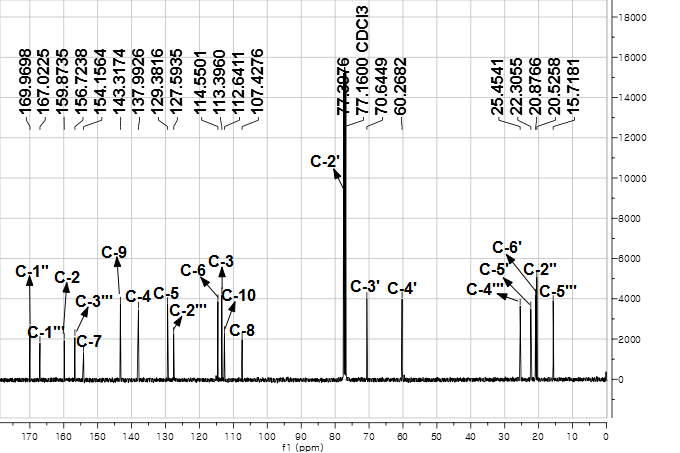


**Figure S2.2.** ^13^C NMR spectrum of .(+) 3'-acetoxy-4'-tigloylkhellacton (**2**).

**
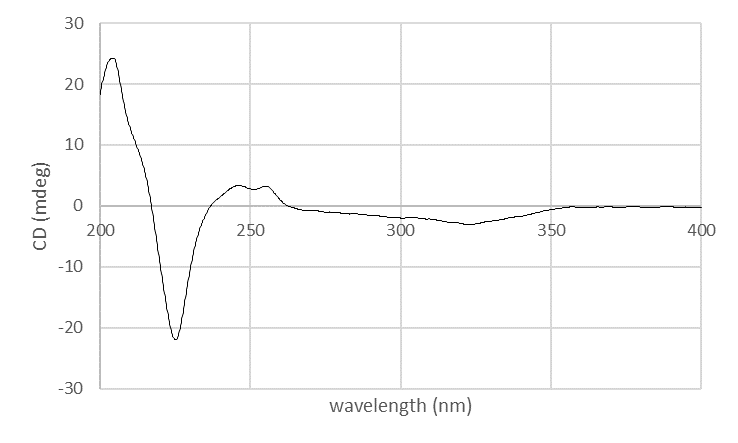
**

**Figure S2.3.** CD spectrum of .(+) 3'-acetoxy-4'-tigloylkhellacton (**2**).
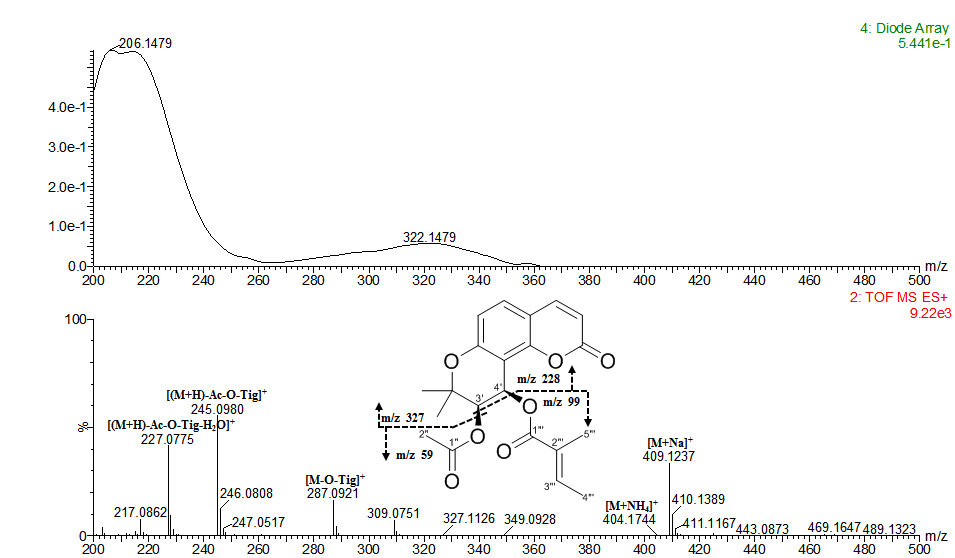


**Figure S2.4.** UV spectrum and MS/MS spectrum of .(+) 3'-acetoxy-4'-tigloylkhellacton (**2**).


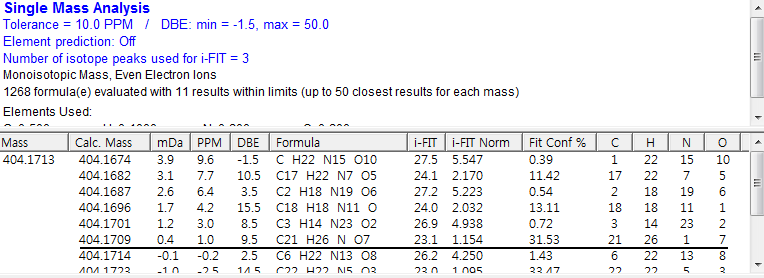


**Figure S2.5.** HR-ESI-MS data of .(+) 3'-acetoxy-4'-tigloylkhellacton (**2**).


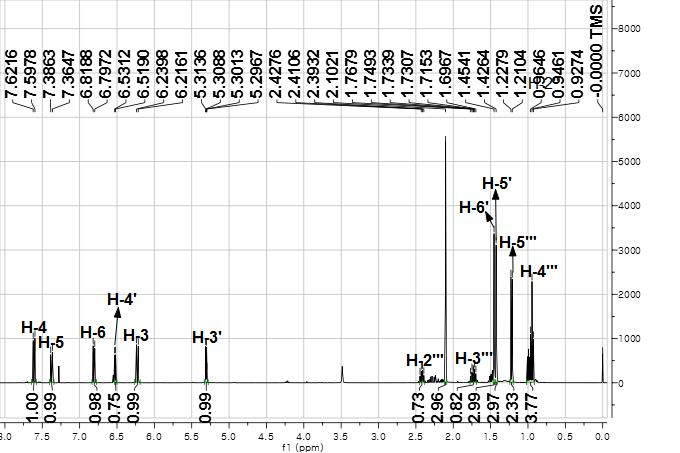


**Figure S3.1.** ^1^H NMR spectrum of (+) 3'-acetoxy-4'-(2-methylbutyroyl)khellactone (**3**).


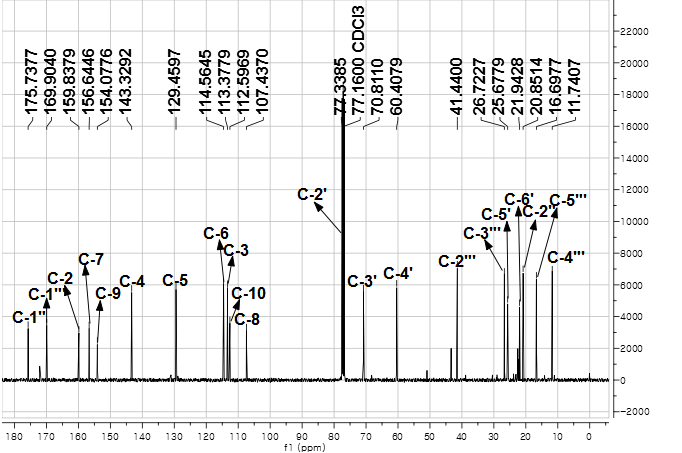


**Figure S3.2.** ^13^C NMR spectrum of (+) 3'-acetoxy-4'-(2-methylbutyroyl)khellactone (**3**).

4'

**
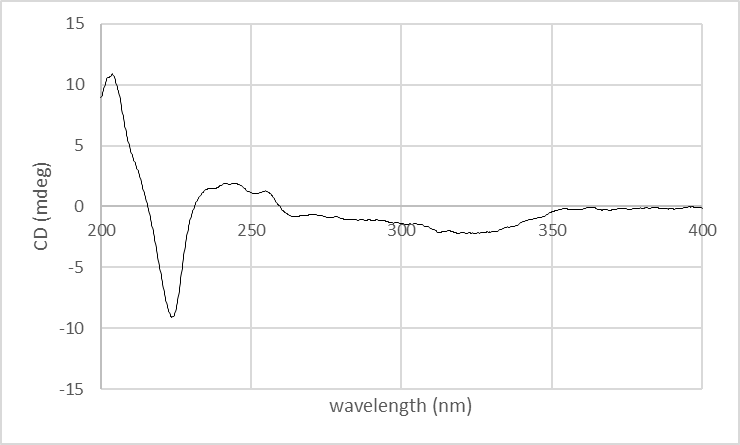
**

**Figure S3.3.** CD spectrum of (+)3'-acetoxy-4'-(2-methylbutyroyl)khellactone (**3**).


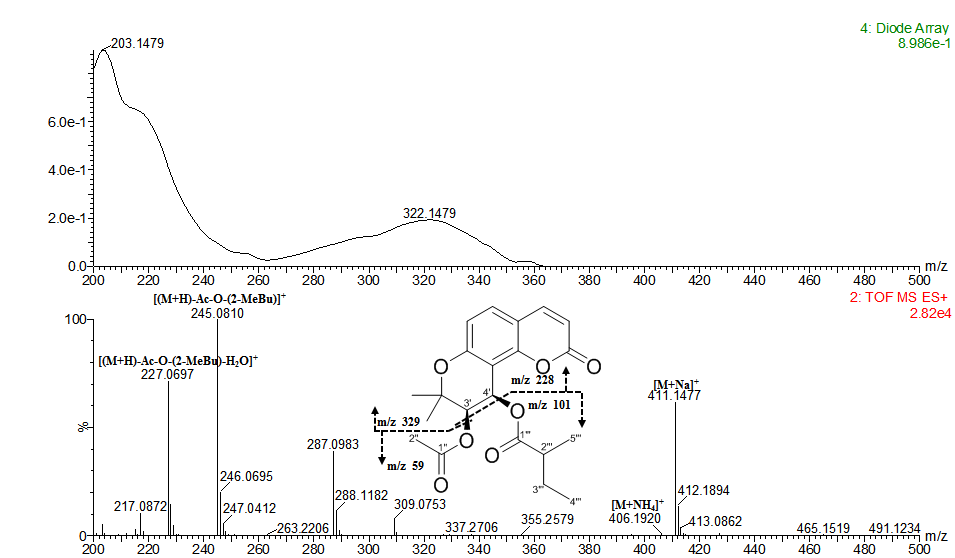


**Figure S3.4.** UV spectrum and MS/MS spectrum of (+)3'-acetoxy-4'-(2-methylbutyroyl) khellactone (**3**).


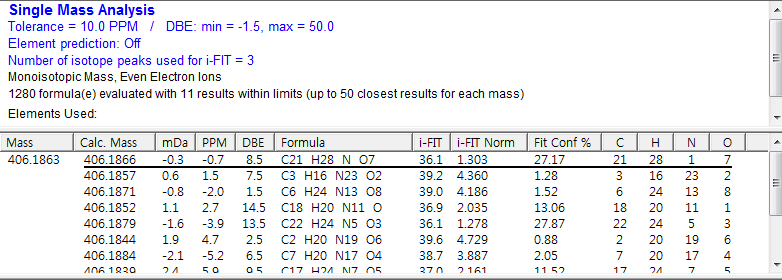


**Figure S3.5.** HR-ESI-MS data of (+)3'-acetoxy-4'-(2-methylbutyroyl)khellactone (**3**).


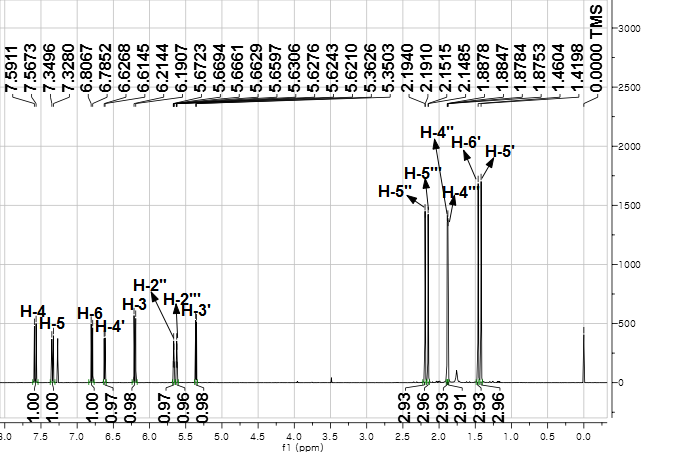


**Figure S4.1.** ^1^H NMR spectrum of (-) 3', 4'-disenecioylkhellactone (**4**).


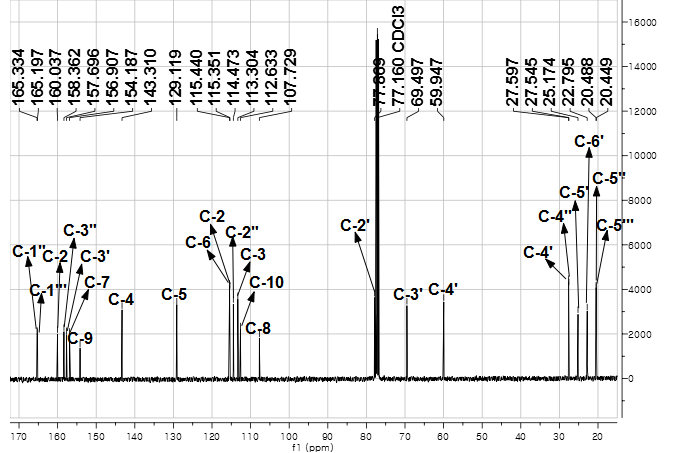


**Figure S4.2.** ^13^C NMR spectrum of (-) 3', 4'-disenecioylkhellactone (**4**).

**
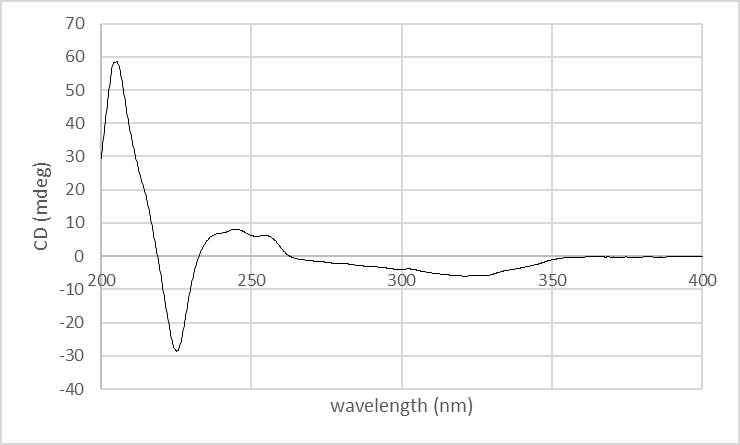
**

**Figure S4.3.** CD spectrum of (-) 3',4'-di senecioylkhellactone (**4**).


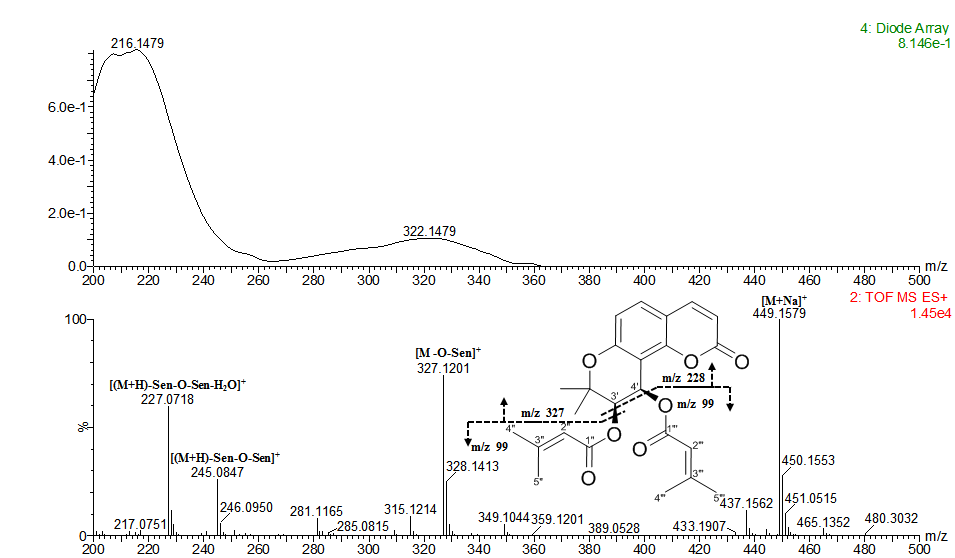


**Figure S4.4.** UV spectrum and MS/MS spectrum of (-) 3',4'-di senecioylkhellactone (**4**).


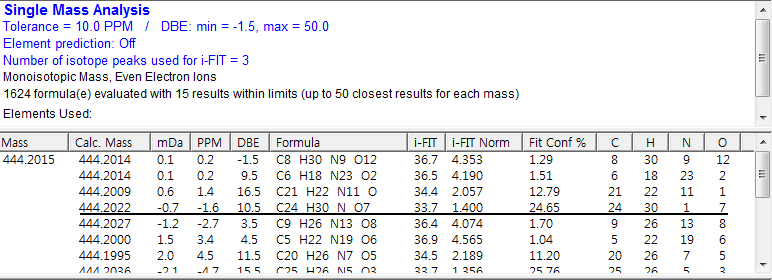


**Figure S4.5.** HR-ESI-MS data of (-) 3',4'-di senecioylkhellactone (**4**).


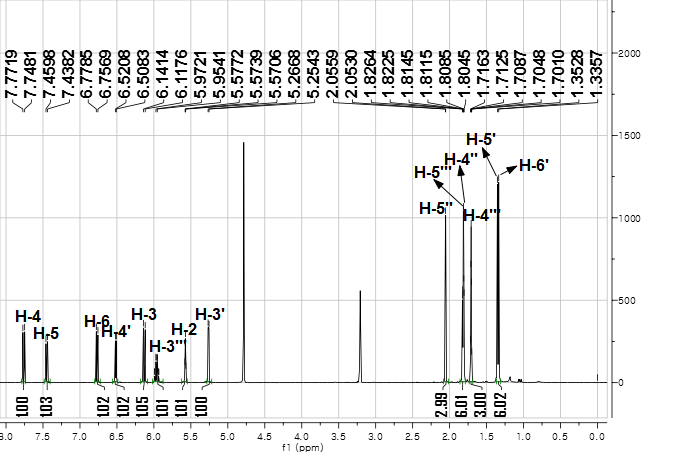


**Figure S5.1.** ^1^H NMR spectrum of (-) 3'-senecioyl-4'-angeloylkhellactone (**5**).


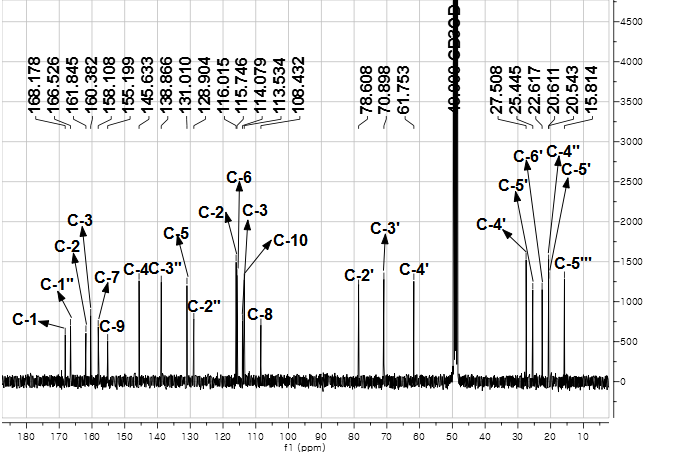
**Figure S5.2.** ^13^C NMR spectrum of (-) 3'-senecioyl-4'-angeloylkhellactone (**5**).

**
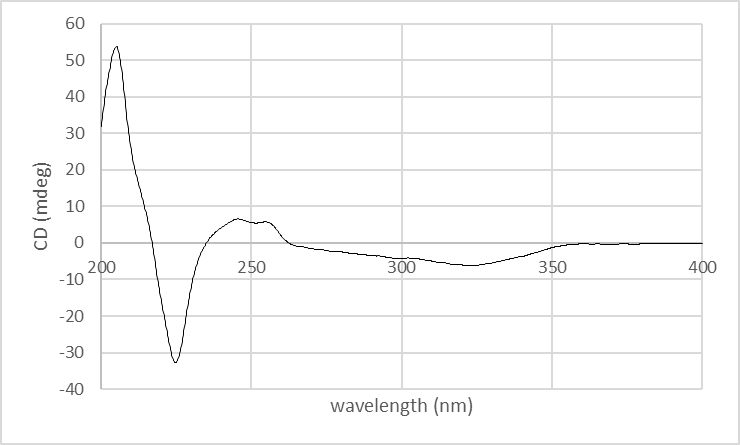
**

**Figure S5.3.** CD spectrum of (-) 3'-senecioyl-4'-angeloylkhellactone (**5**).


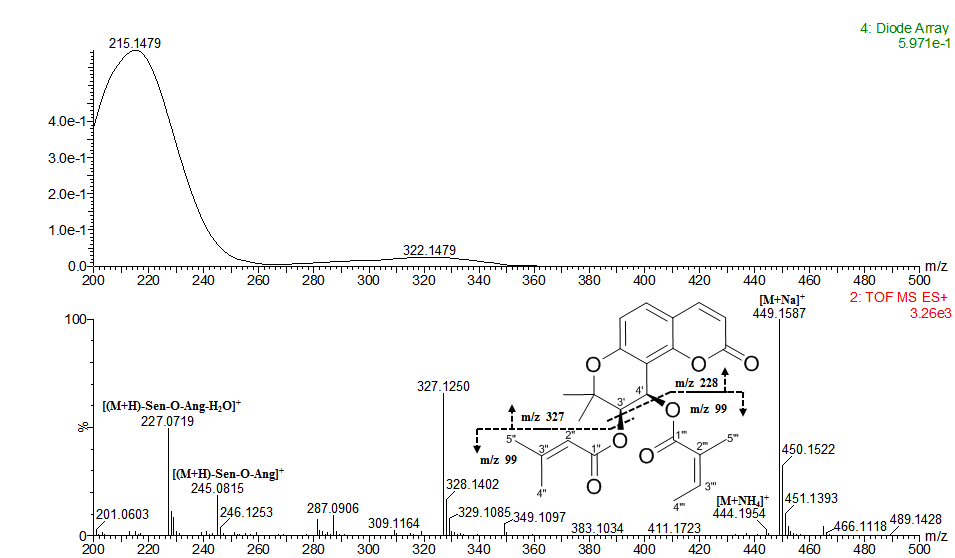


**Figure S5.4.** UV spectrum and MS/MS spectrum of (-) 3'-senecioyl-4'-angeloylkhellactone (**5**).


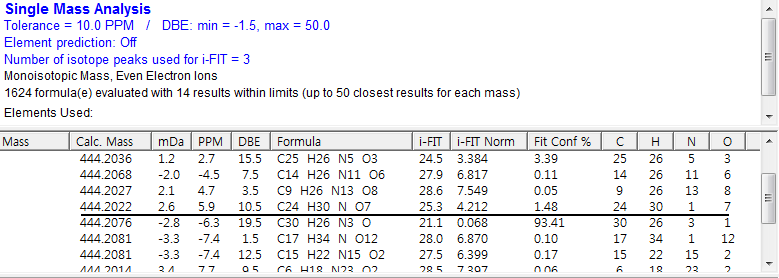


**Figure S5.5.** HR-ESI-MS data of (-) 3'-senecioyl-4'-angeloylkhellactone (**5**).

**
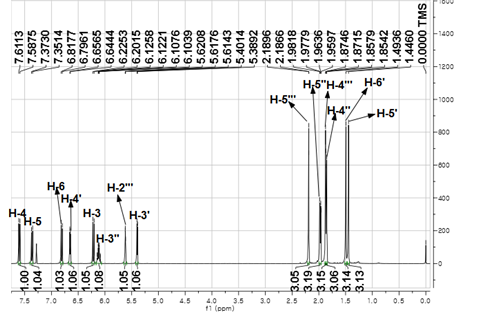
Figure S6.1.** ^1^H NMR spectrum of (+) 3'-angeloyl-4'-senecioylkhellactone (**6**).

**
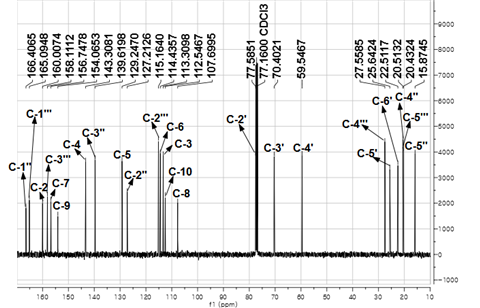
Figure S6.2.** ^13^C NMR spectrum of (+) 3'-angeloyl-4'-senecioylkhellactone (**6**).


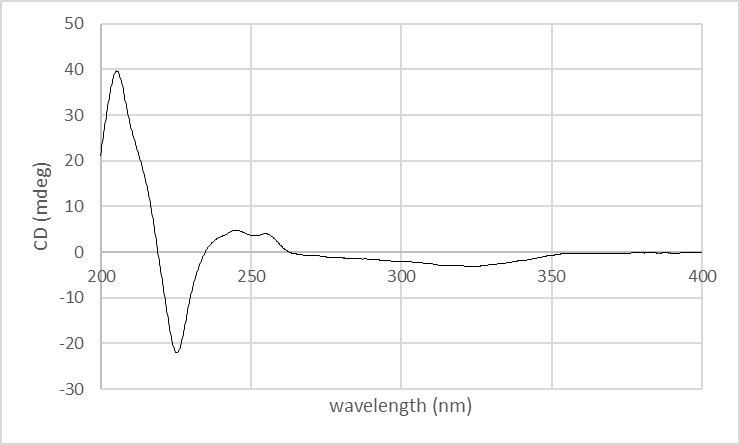


**Figure S6.3.** CD spectrum of (+) 3'-angeloyl-4'-senecioylkhellactone (**6**).


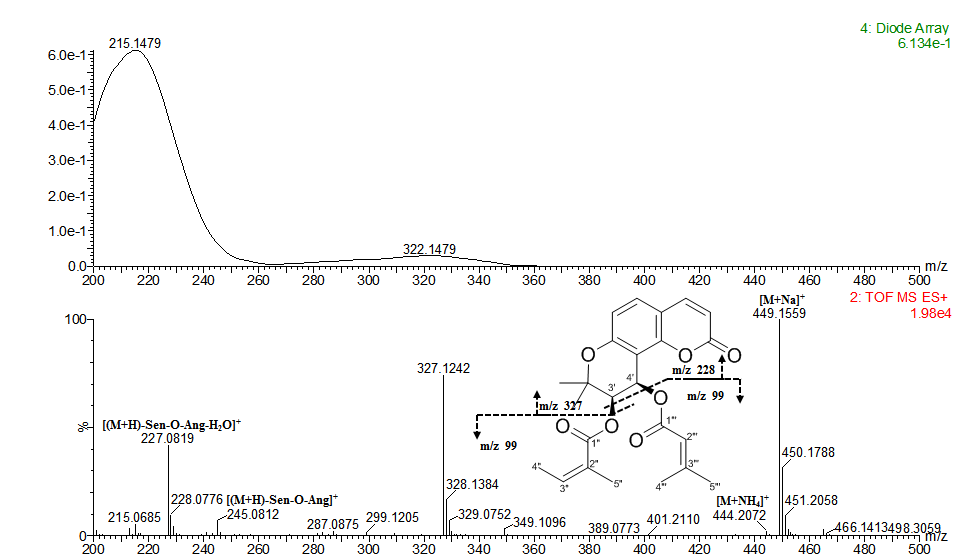


**Figure S6.4.** UV spectrum and MS/MS spectrum of (+) 3'-angeloyl-4'-senecioylkhellactone (**6**).


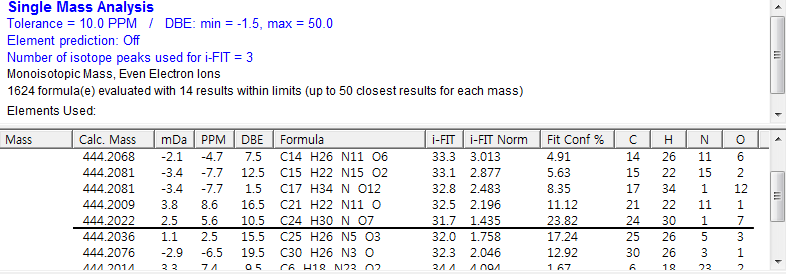


**Figure S6.5.** HR-ESI-MS data of (+) 3'-angeloyl-4'-senecioylkhellactone (**6**).

**
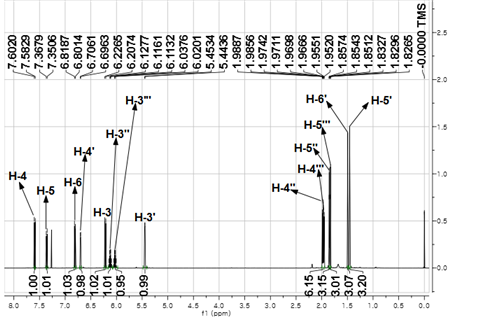
Figure S7.1.** ^1^H NMR spectrum of (+) 3', 4'-diangeloylkhellactone (**7**).

**
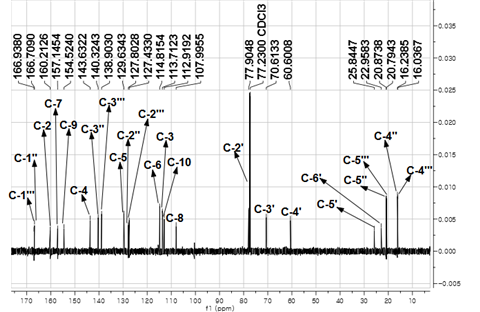
Figure S7.2.** ^13^C NMR spectrum of (+) 3', 4'-diangeloylkhellactone (**7**).


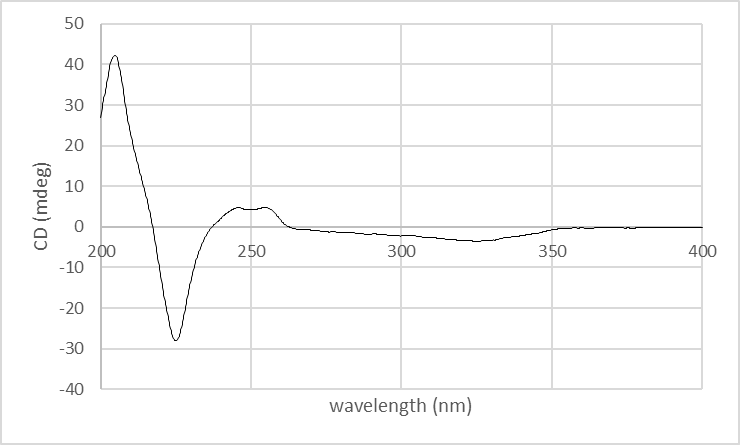


**Figure S7.3.** CD spectrum of (+) 3', 4'-diangeloylkhellactone (**7**).


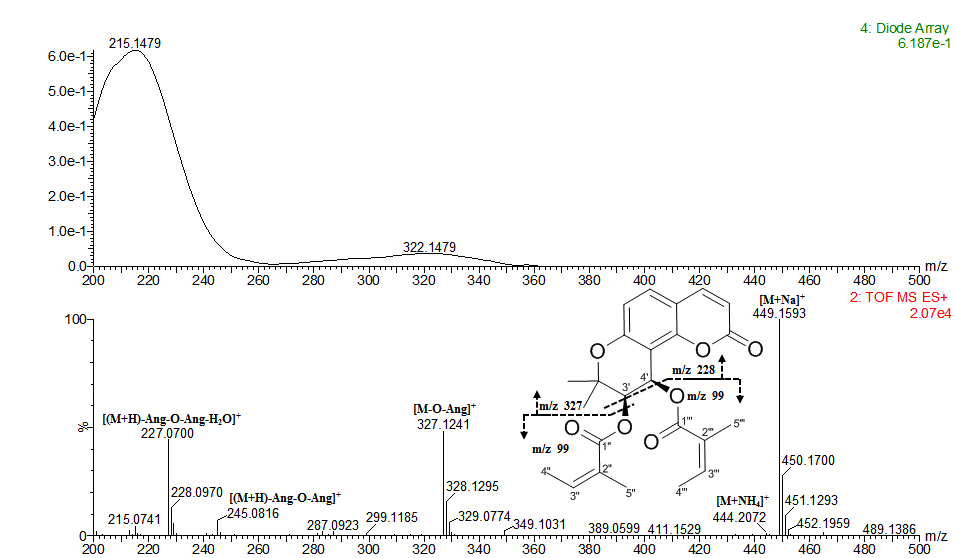


**Figure S7.4.** UV spectrum and MS/MS spectrum of (+) 3', 4'-diangeloylkhellactone (**7**).


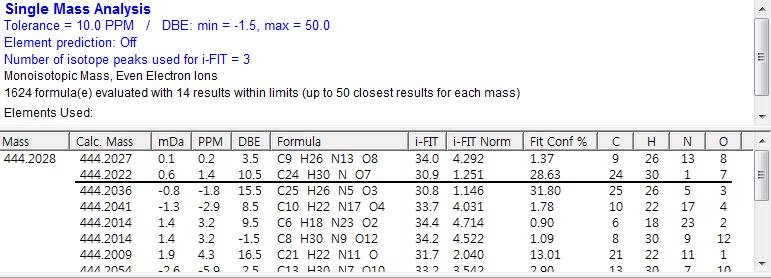


**Figure S7.5.** HR-ESI-MS data of (+) 3', 4'-diangeloylkhellactone (**7**).

**
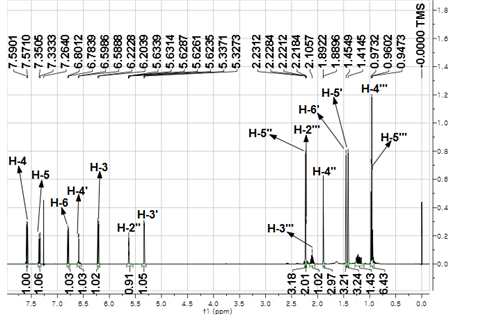
Figure S8.1.** ^1^H NMR spectrum of (-) 3'-senecioyl-4'-isovalerylkhellactone (**8**).

**
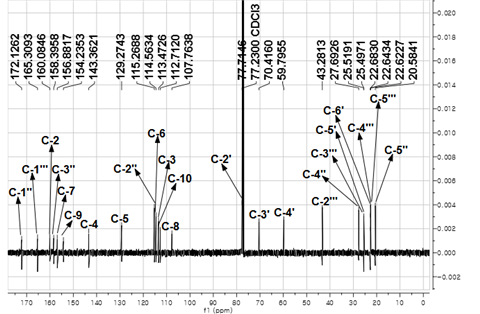
Figure S8.2.** ^13^C NMR spectrum of (-) 3'-senecioyl-4'-isovalerylkhellactone (**8**).


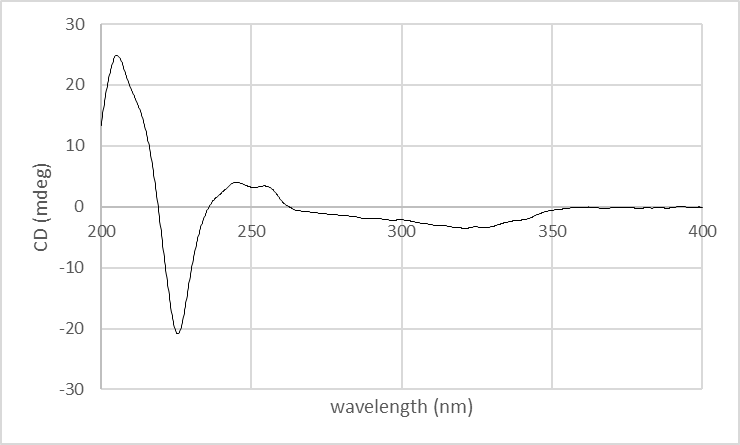


**Figure S8.3.** CD spectrum of (-) 3'-senecioyl-4'-isovalerylkhellactone (**8**).


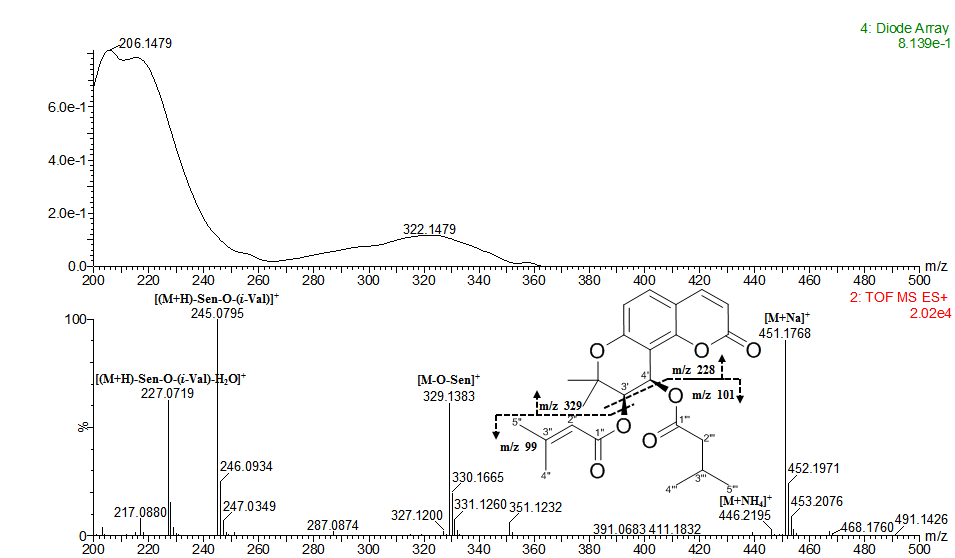


**Figure S8.4.** UV spectrum and MS/MS spectrum of (-) 3'-senecioyl-4'-isovalerylkhellactone (**8**).


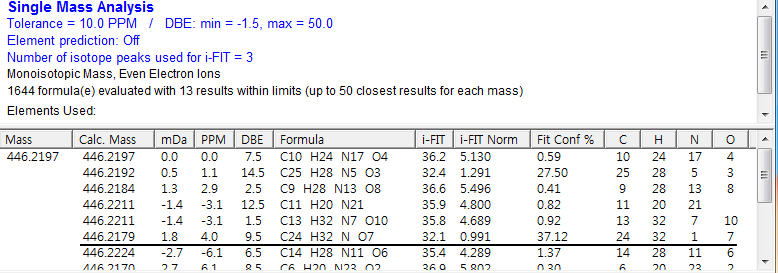


**Figure S8.5.** HR-ESI-MS data of (-) 3'-senecioyl-4'-isovalerylkhellactone (**8**).

**
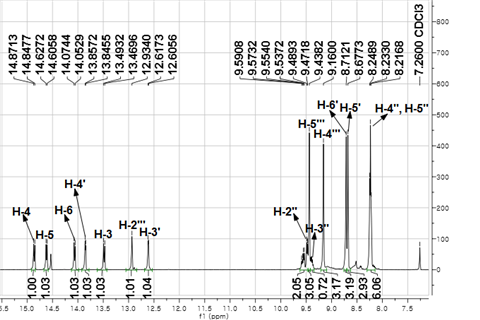
Figure S9.1.** ^1^H NMR spectrum of (-) 3'-isovaleryl-4'-senecioylkhellactone (**9**).

**
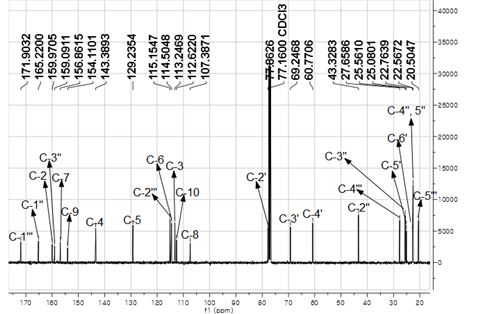
Figure S9.2.** ^13^C NMR spectrum of (-) 3'-isovaleryl-4'-senecioylkhellactone (**9**).


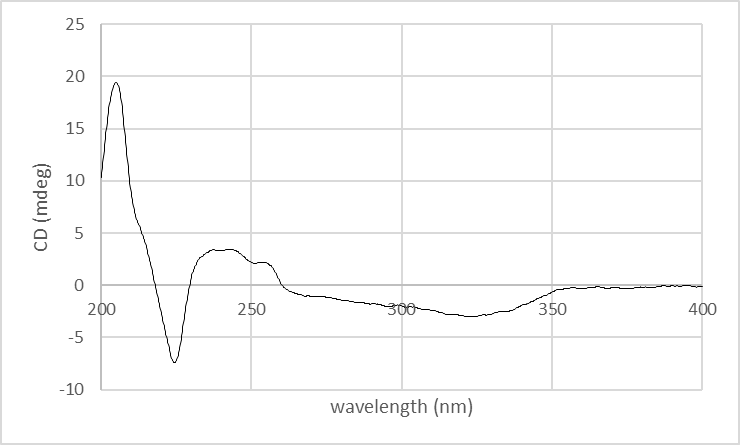


**Figure S9.3.** CD spectrum of (-) 3'-isovaleryl-4'-senecioylkhellactone (**9**).


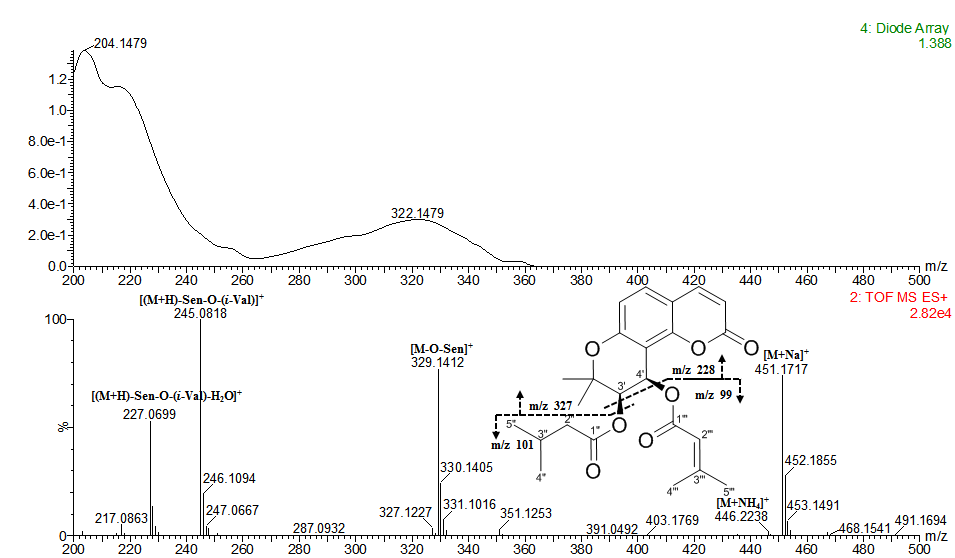


**Figure S9.4.** UV spectrum and MS/MS spectrum of (-) 3'-isovaleryl-4'-senecioylkhellactone (**9**).


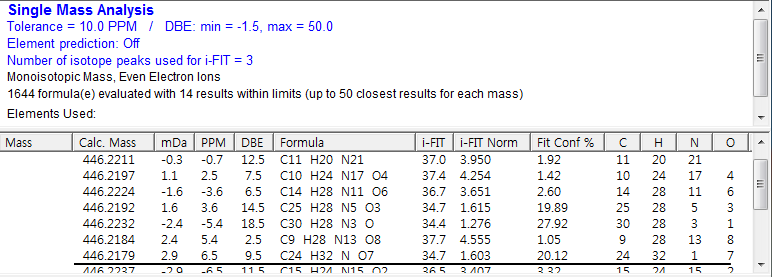


**Figure S9.5.** HR-ESI-MS data of (-) 3'-isovaleryl-4'-senecioylkhellactone (**9**).

**
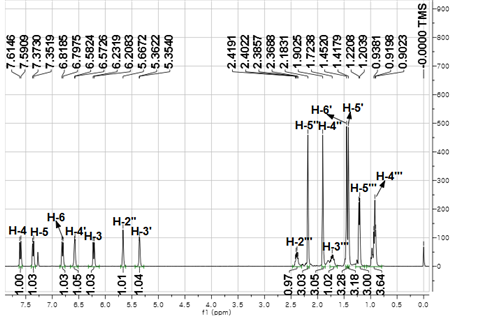
Figure S10.1.** ^1^H NMR spectrum of (-) 3'-senecioyl-4'-(2-methylbutyroyl) khellactone (**10**) .

**
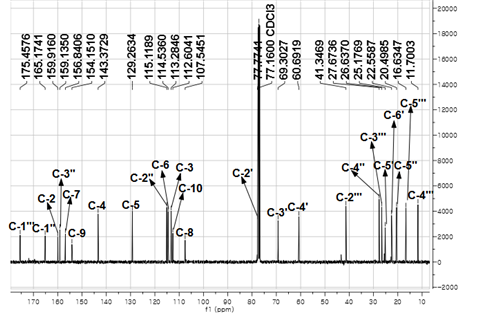
Figure S10.2.** ^13^C NMR spectrum of (-) 3'-senecioyl-4'-(2-methylbutyroyl) khellactone (**10**).

**
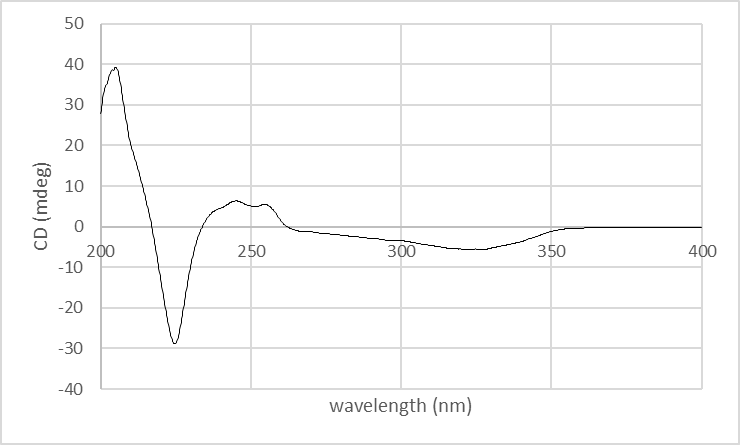
**

**Figure S10.3.** CD spectrum of (-) 3'-senecioyl-4'-(2-methylbutyroyl) khellactone (**10**).


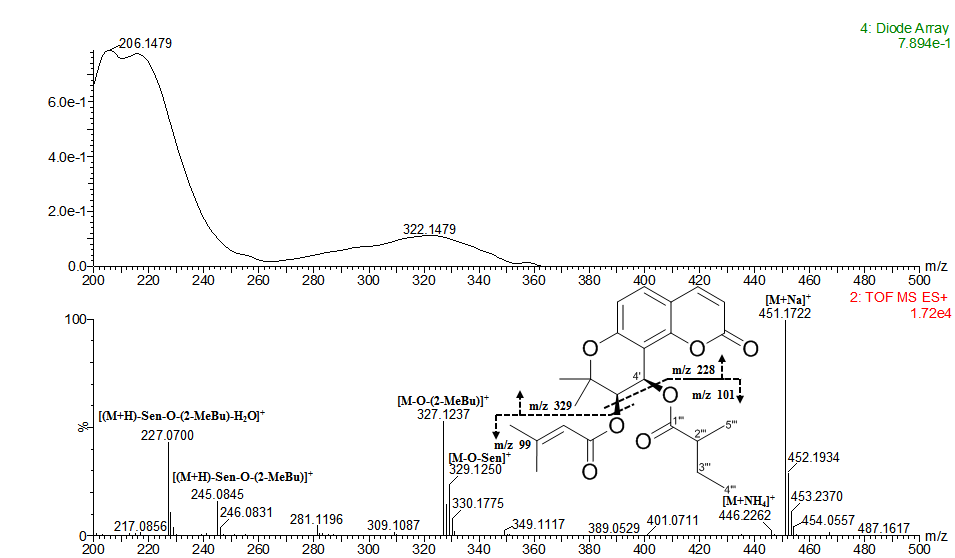


**Figure S10.4.** UV spectrum and MS/MS spectrum of (-) 3'-senecioyl-4'-(2-methylbutyroyl) khellactone (**10**).


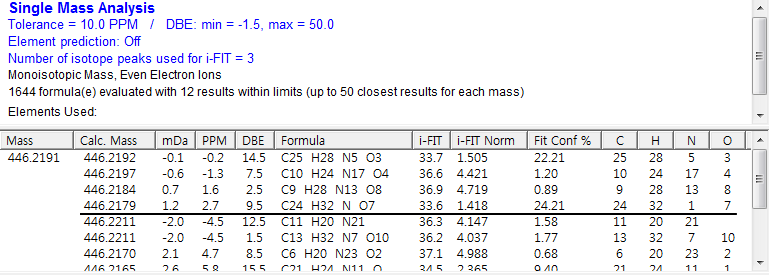


**Figure S10.5.** HR-ESI-MS data of (-) 3'-senecioyl-4'-(2-methylbutyroyl) khellactone (**10**).


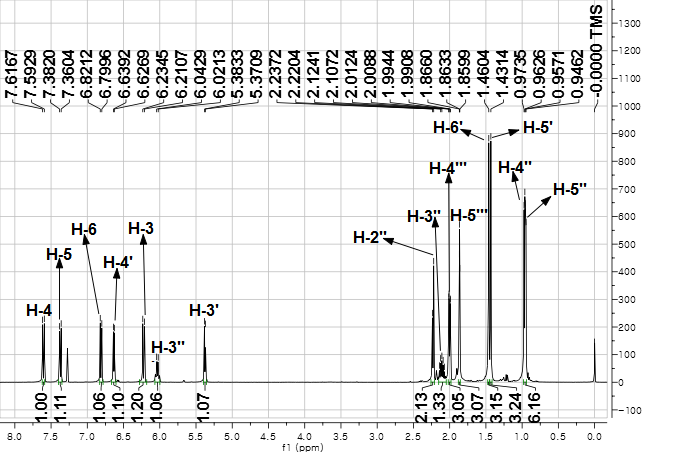


**Figure S11.1.** ^1^H NMR spectrum of (-) 3'-isovaleryl-4'-angeloylkhellactone (**11**).


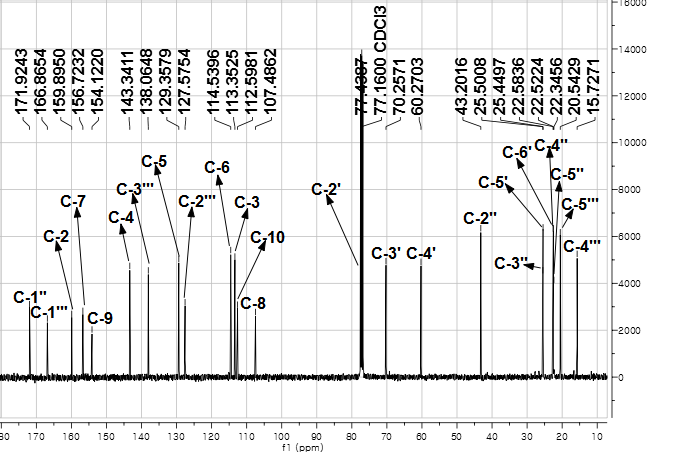


**Figure S11.2.** ^13^C NMR spectrum of (-) 3'-isovaleryl-4'-angeloylkhellactone (**11**).


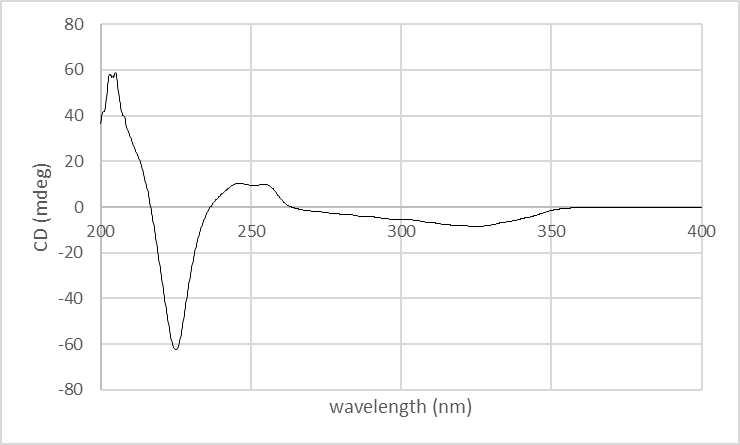


**Figure S11.3.** CD spectrum of (-) 3'-isovaleryl-4'-angeloylkhellactone (**11**).


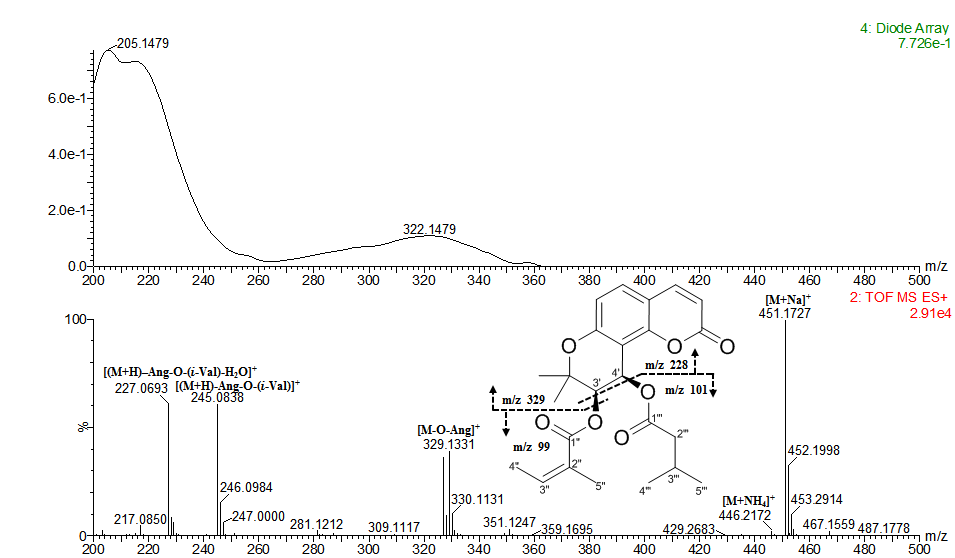


**Figure S11.4.** UV spectrum and MS/MS spectrum of (-) 3'-isovaleryl-4'-angeloylkhellactone (**11**).


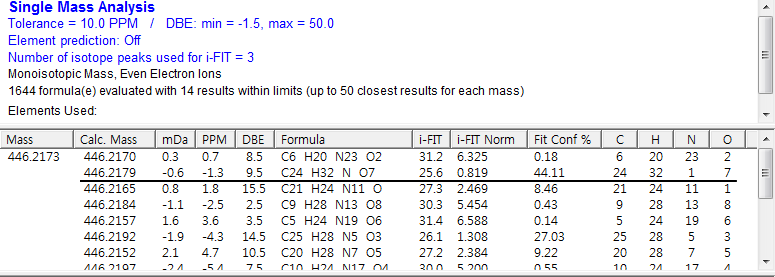


**Figure S11.5.** HR-ESI-MS data of (-) 3'-isovaleryl-4'-angeloylkhellactone (**11**).


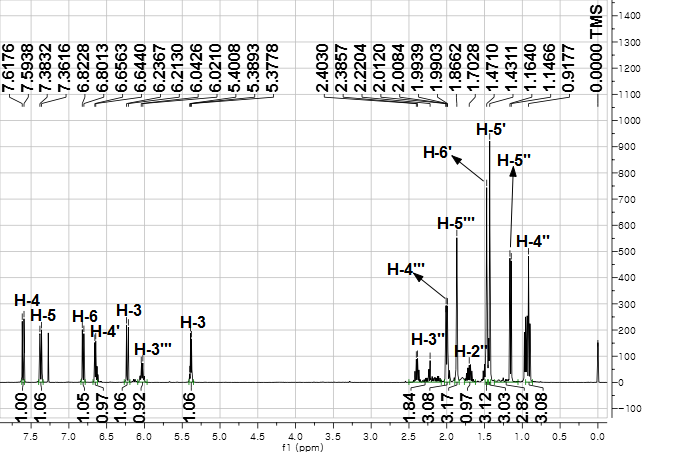


**Figure S12.1.** ^1^H NMR spectrum of (-) 3'-(2-methylbutyryl)-4'-angeloylkhellactone (**12**).


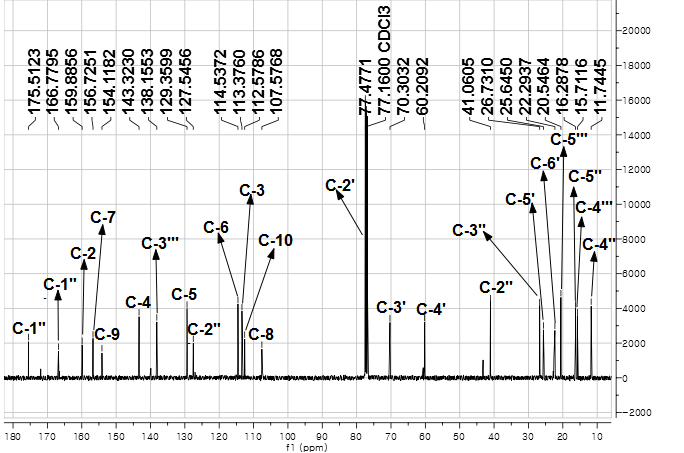


**Figure S12.2.** ^13^C NMR spectrum of (-) 3'-(2-methylbutyryl)-4'-angeloylkhellactone (**12**).


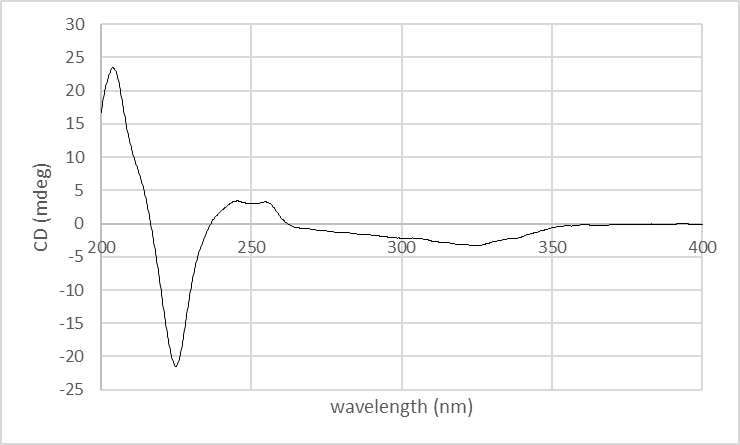


**Figure S12.3.** CD spectrum of (-) 3'-(2-methylbutyryl)-4'-angeloylkhellactone (**12**) .


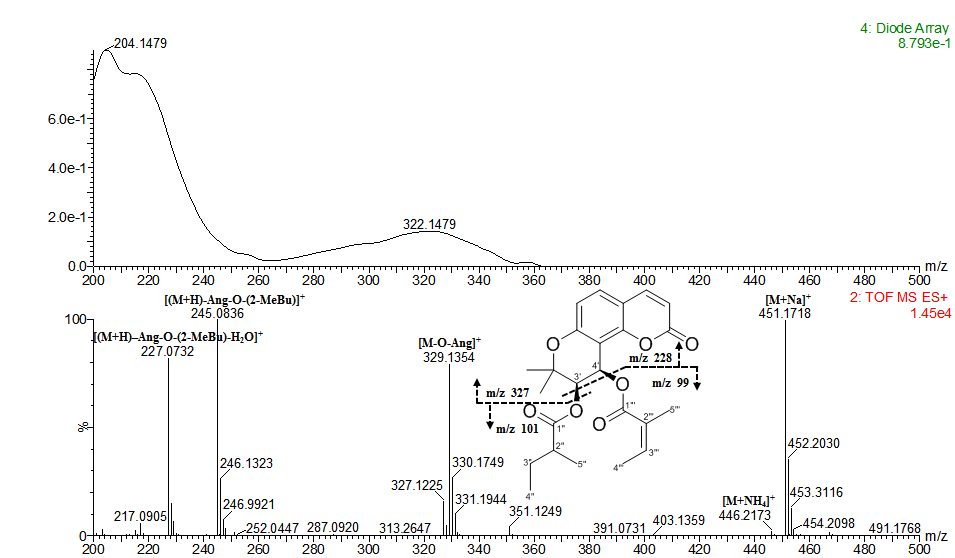


**Figure S12.4.** UV spectrum and MS/MS spectrum of (-) 3'-(2-methylbutyryl)-4'-angeloylkhellactone (**12**).


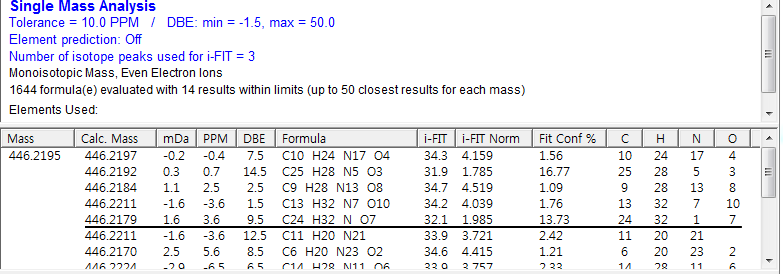


**Figure S12.5.** HR-ESI-MS data of (-) 3'-(2-methylbutyryl)-4'-angeloylkhellactone (**12**).

.


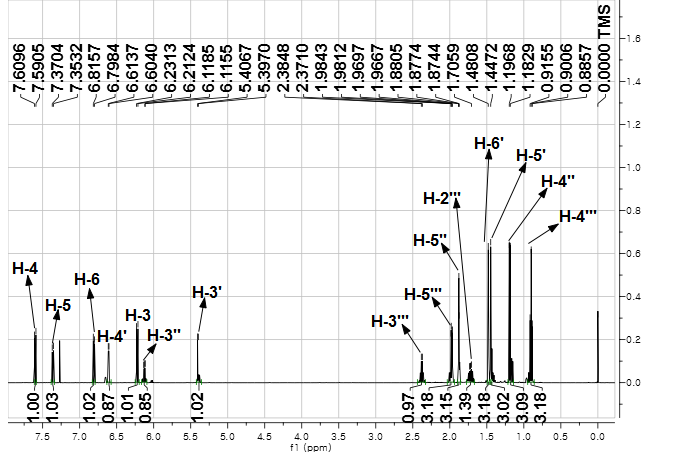


**Figure S13.1.** ^1^H NMR spectrum of (+) 3'-angeloyl-4'-(2-methylbutyryl)khellactone (**13**).


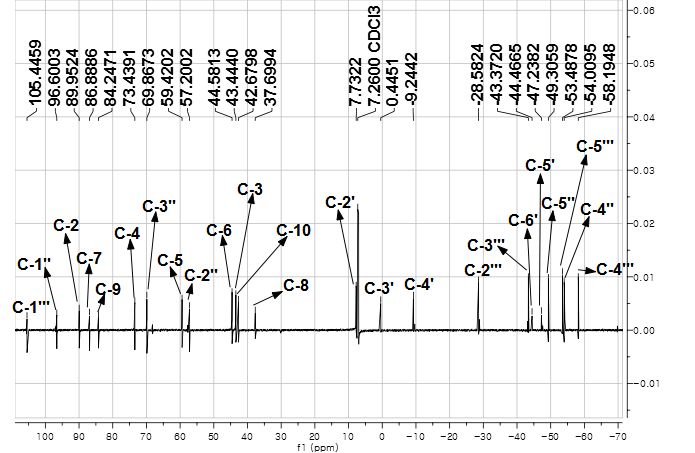


**Figure S13.2.** ^13^C NMR spectrum of (+) 3'-angeloyl-4'-(2-methylbutyryl)khellactone (**13**).


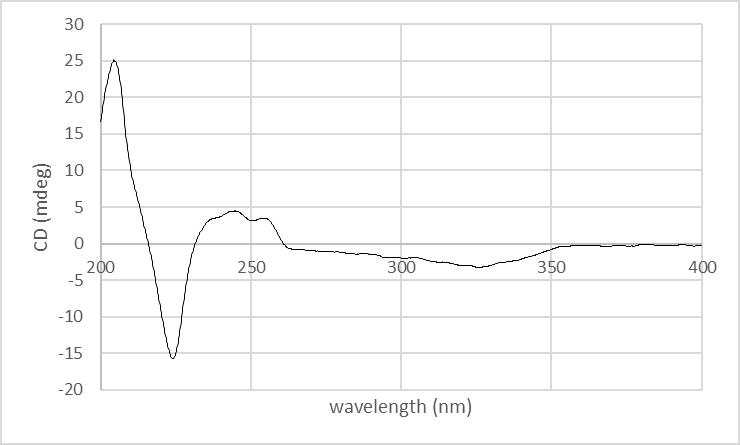


**Figure S13.3.** CD spectrum of (+) 3'-angeloyl-4'-(2-methylbutyryl)khellactone (**13**).


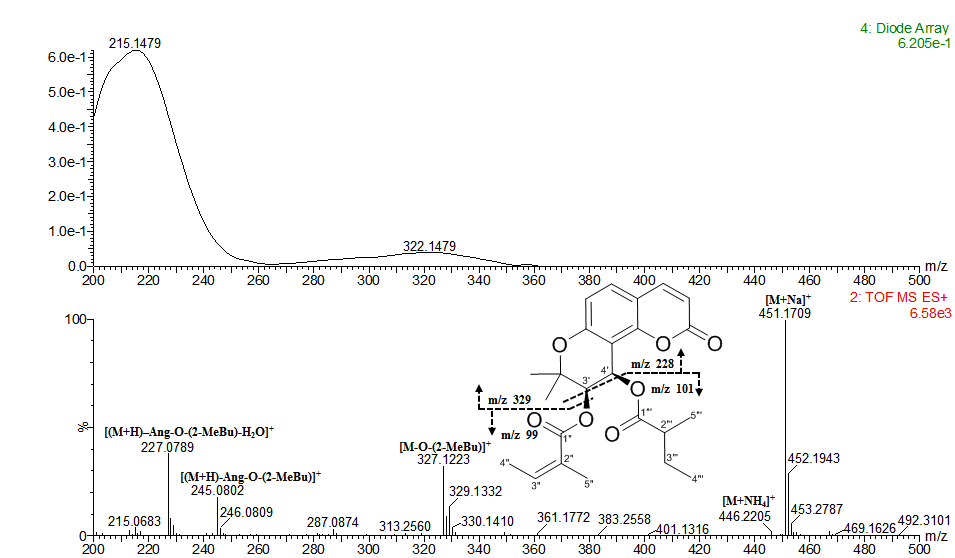
**Figure S13.4.** UV spectrum and MS/MS spectrum of (+) 3'-angeloyl-4'-(2-methylbutyryl)khellactone (**13**).


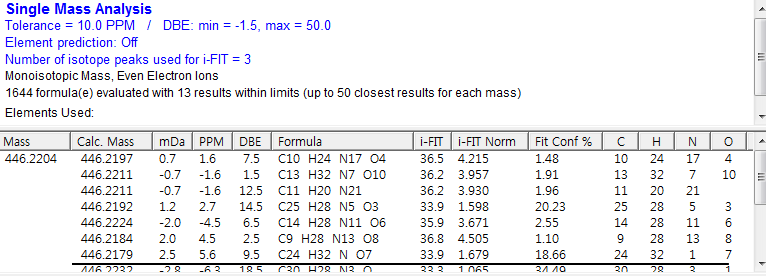


**Figure S13.5.** HR-ESI-MS data of (+) 3'-angeloyl-4'-(2-methylbutyryl)khellactone (**13**).


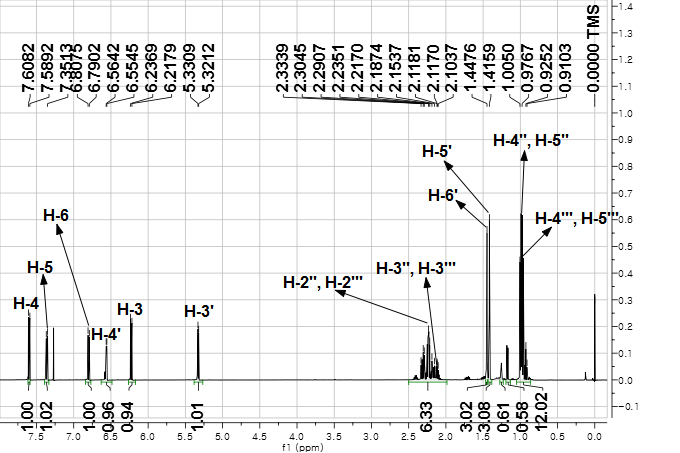


**Figure S14.1.** ^1^H NMR spectrum of (-) 3',4'-diisovalerylkhellactone (**14**).


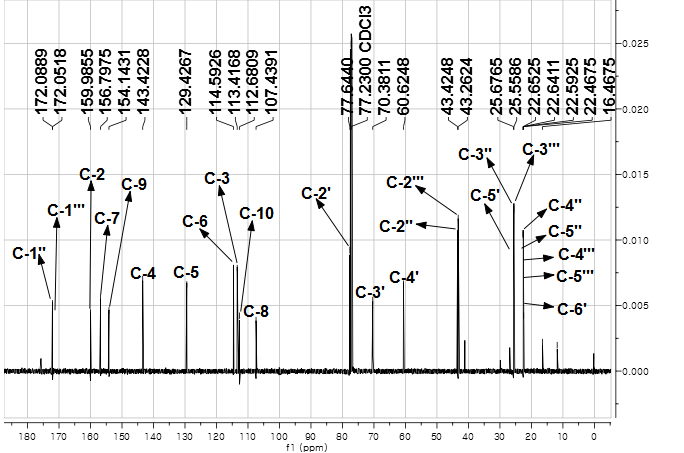


**Figure S14.2.** ^13^C NMR spectrum of (-) 3',4'-diisovalerylkhellactone (**14**).


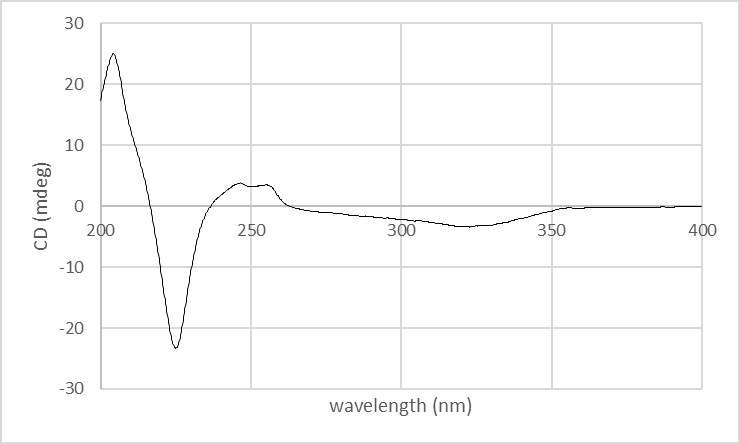


**Figure S14.3** CD spectrum of (-) 3',4'-diisovalerylkhellactone (**14**).


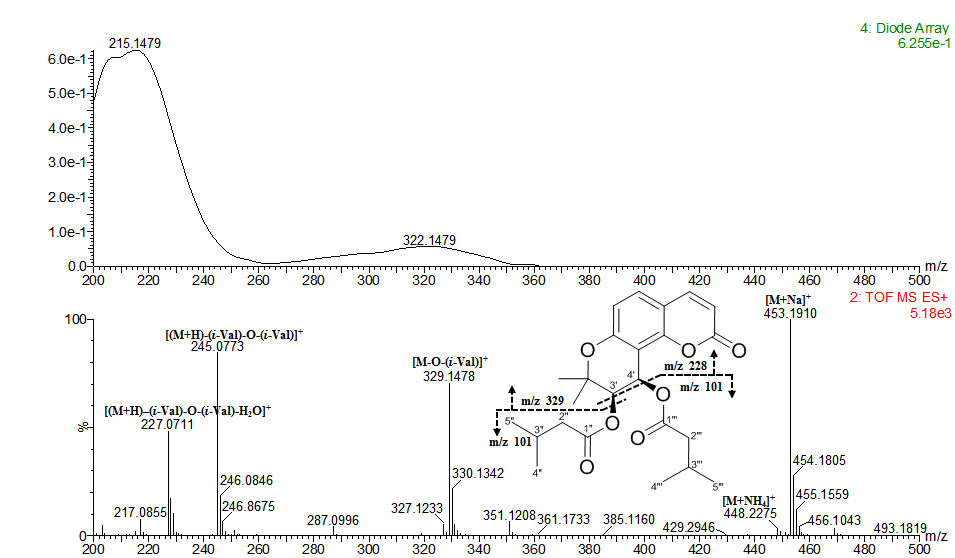


**Figure S14.4.** UV spectrum and MS/MS spectrum of (-) 3',4'-diisovalerylkhellactone (**14**).


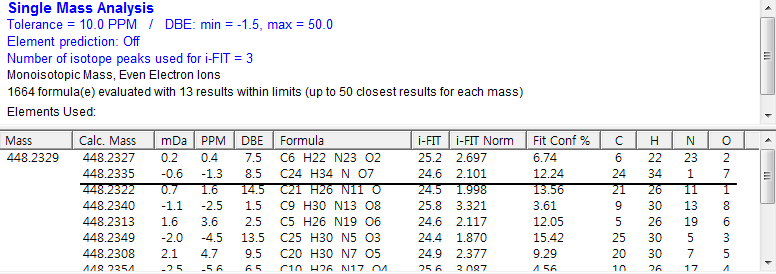


**Figure S14.5.** HR-ESI-MS data of (-) 3',4'-diisovalerylkhellactone (**14**).


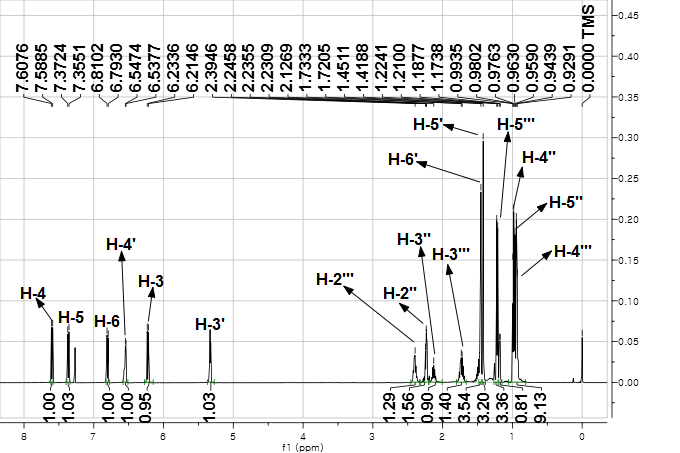


**Figure S15.1.** ^1^H NMR spectrum of (-) 3'-isovaleryl-4'-(2-methylbutyryl)khellactone (**15**).


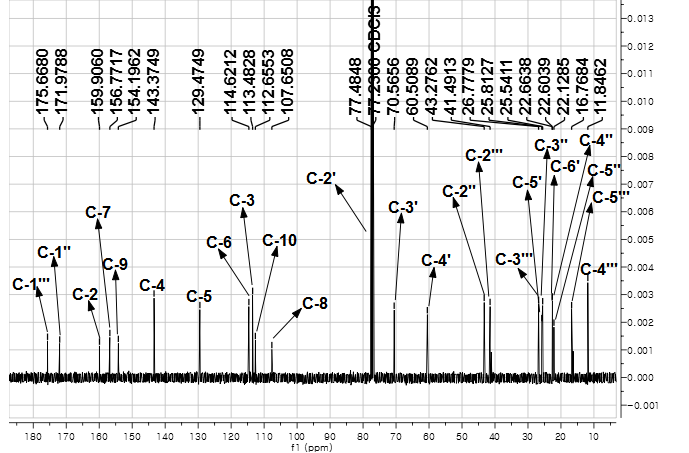


**Figure S15.2.** ^13^C NMR spectrum of (-) 3'-isovaleryl-4'-(2-methylbutyryl)khellactone (**15**).


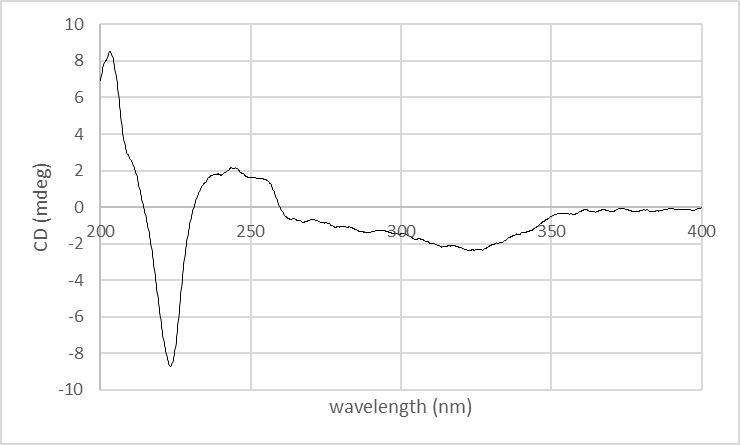


**Figure S15.3.** CD spectrum of (-) 3'-isovaleryl-4'-(2-methylbutyryl)khellactone (**15**).


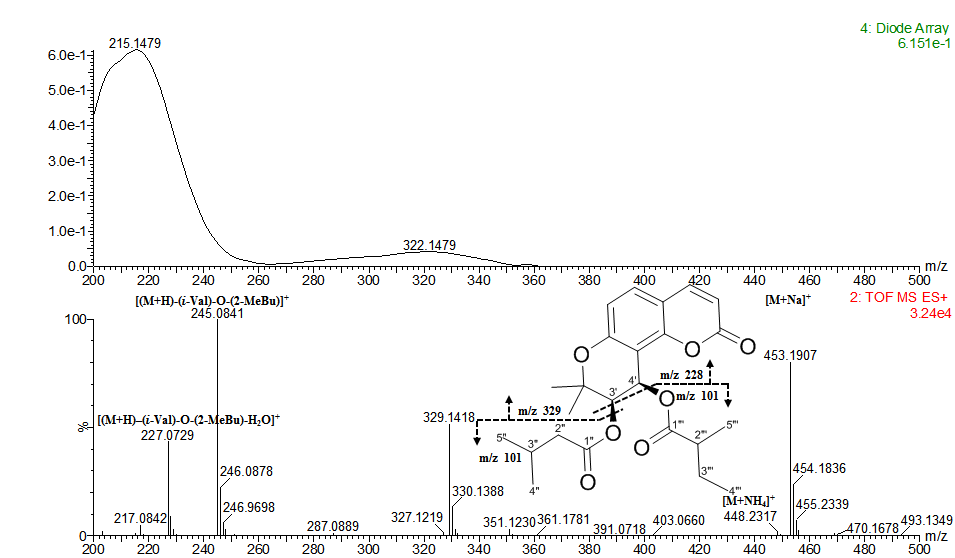


**Figure S15.4.** UV spectrum and MS/MS spectrum of (-) 3'-isovaleryl-4'-(2-methylbutyryl)khellactone (**15**).


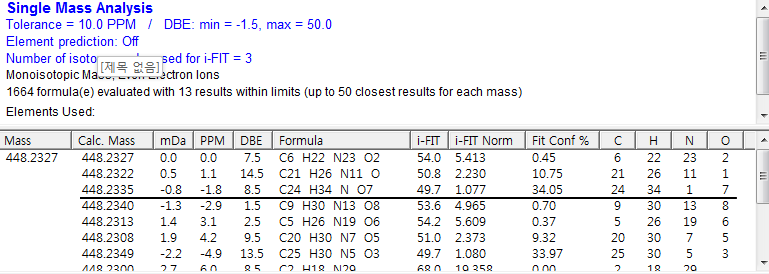


**Figure S15.5.** HR-ESI-MS data of (-) 3'-isovaleryl-4'-(2-methylbutyryl)khellactone (**15**).


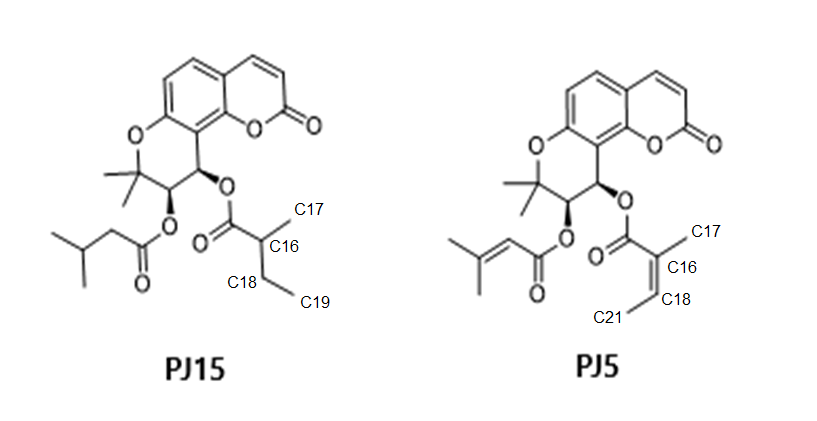


**Figure S16.** Structures of **PJ15** and **PJ5** with the carbon numbers of 2-methyl-butane and 2-methyl-butene group, respectively.

(A)


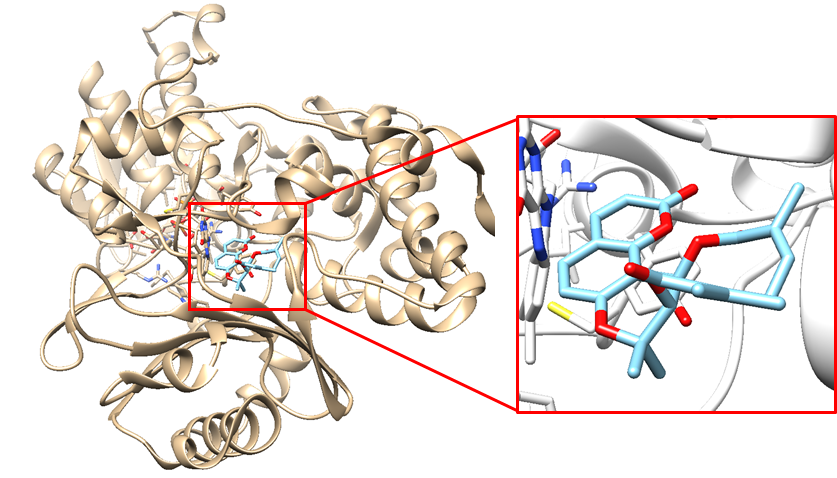


(B)


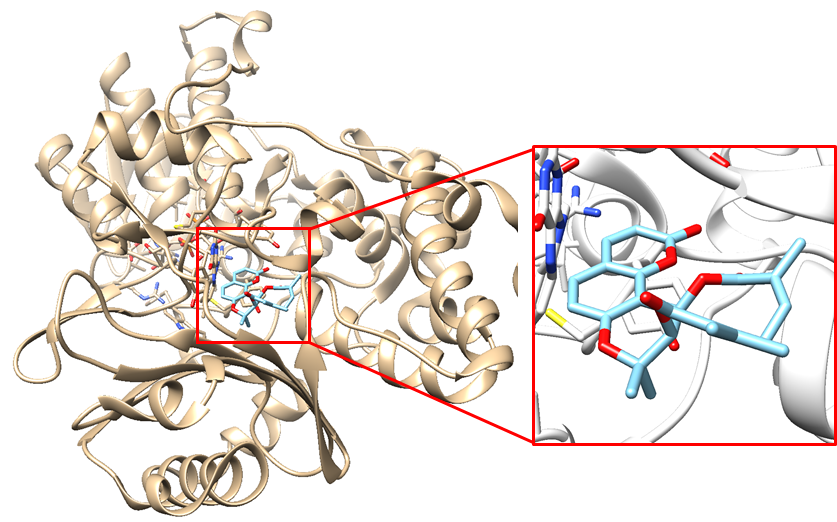


(C)


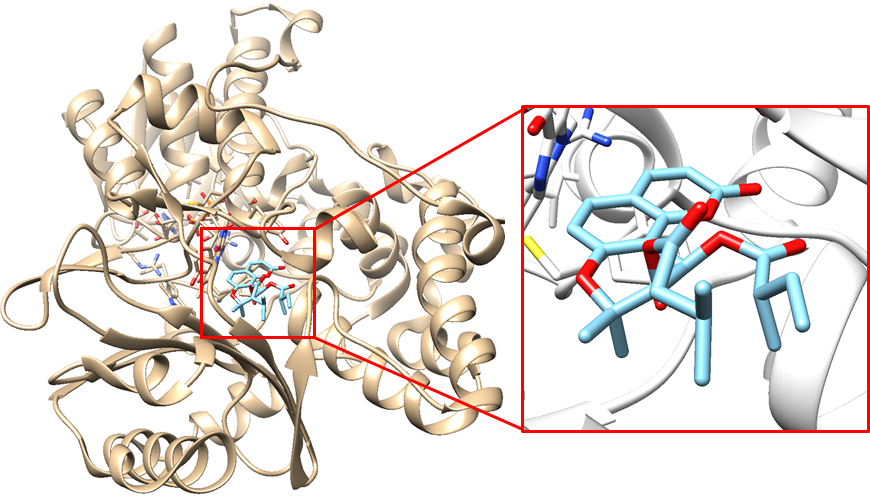


(D)


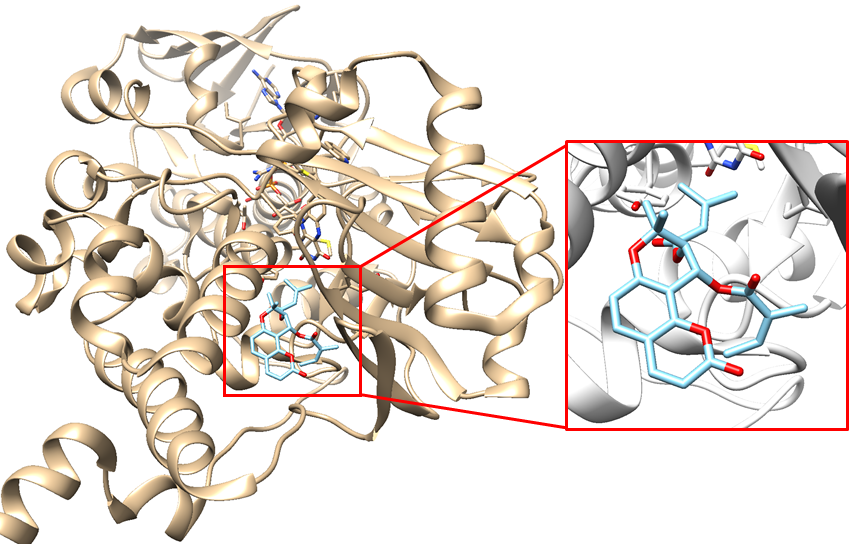


(E)


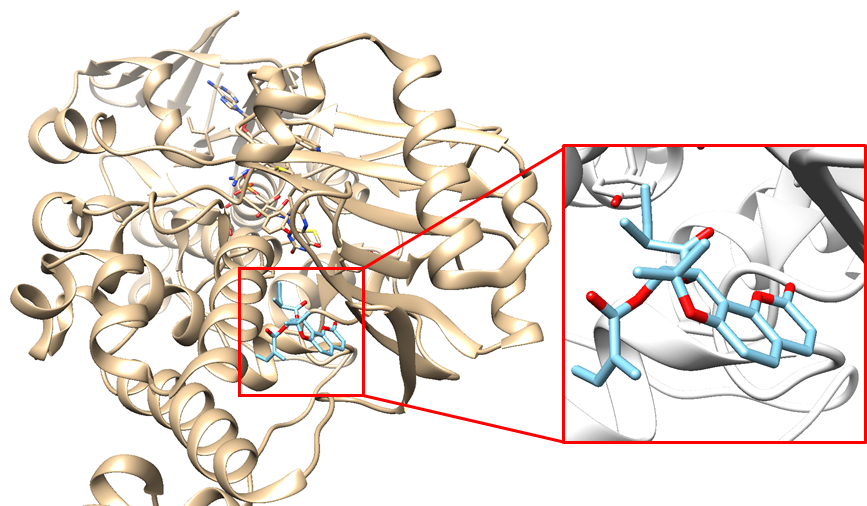


(F)


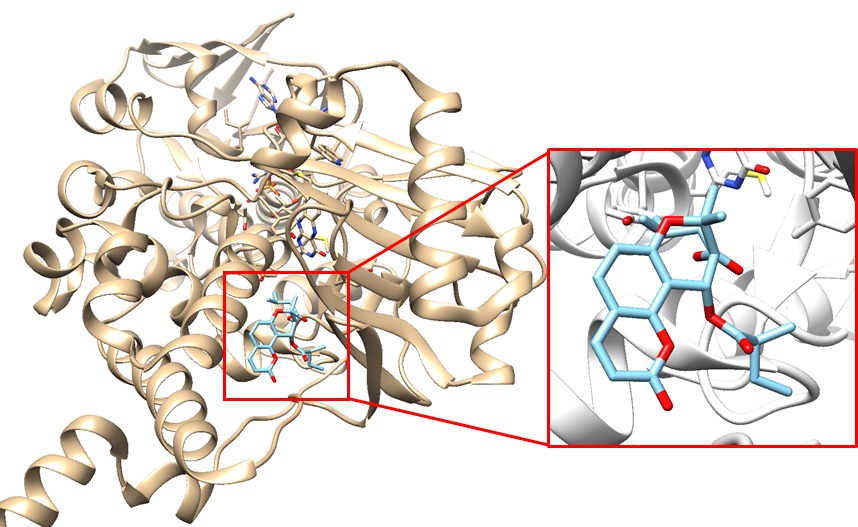


**Figure S17.** Docking simulations of **PJ5**, **PJ13**, and **PJ15** with MAO-A (2Z5X) (A-C, respectively) and MAO-B (4A79) (D-F, respectively).

(A)


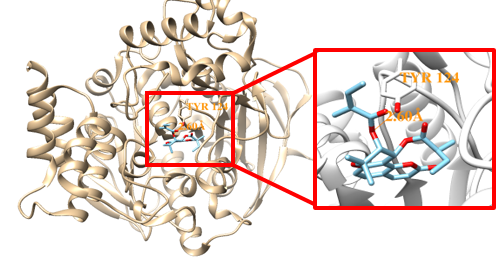


(B)


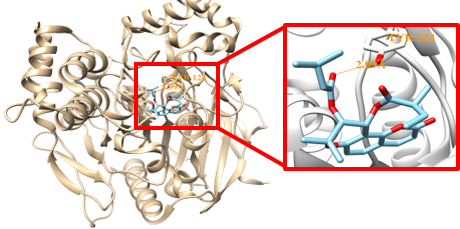


(C)


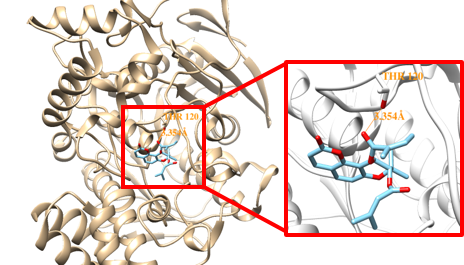


**Figure S18**. Docking simulations of **PJ13** (A) and **PJ15** (B) with AChE (PDB ID: 6O4W) and **PJ5** (C) with BChE (PDB ID: 6QAA) pre-defined with donepezil.

(A)


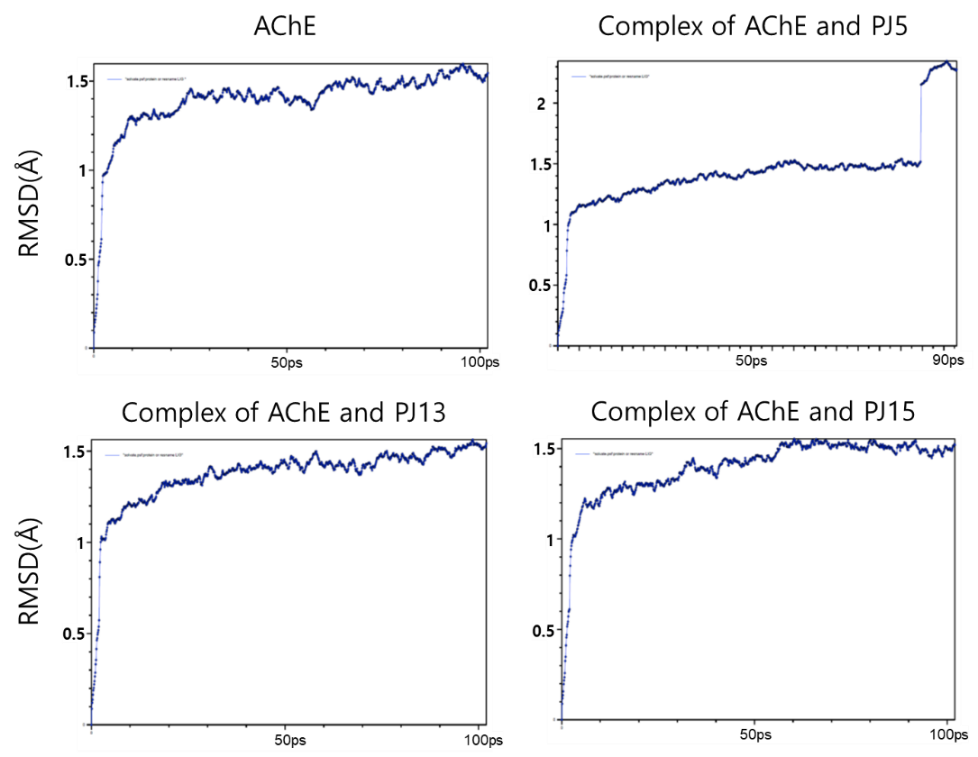


(B)


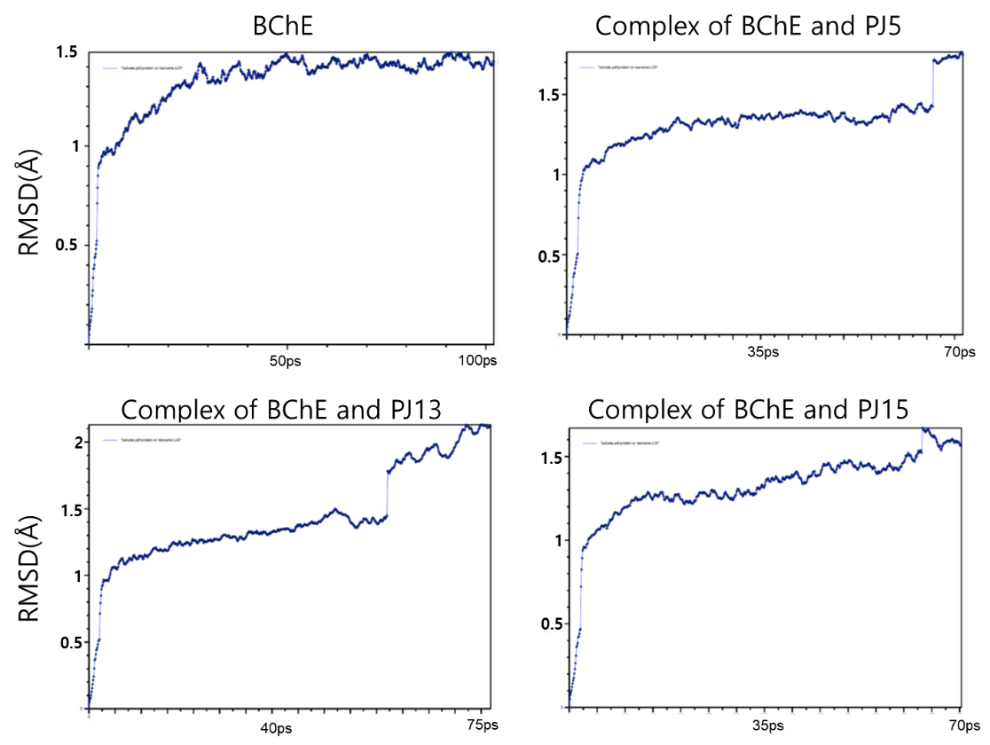


**Figure S19**. Plots of root mean square deviation during 100 ps MD simulation of AChE (A) BChE (B) in complexes with **PJ5**, **PJ13**, and **PJ15**.
